# Distributed Quantization for Partially Cooperating Sensors Using the Information Bottleneck Method

**DOI:** 10.3390/e24040438

**Published:** 2022-03-22

**Authors:** Steffen Steiner, Abdulrahman Dayo Aminu, Volker Kuehn

**Affiliations:** 1 Institute of Communications Engineering, University of Rostock, 18119 Rostock, Germany; volker.kuehn@uni-rostock.de; 2 Pydro GmbH, 18119 Rostock, Germany; aminuabdulrahmandayo@gmail.com

**Keywords:** distributed compression, chief executive officer problem, cooperating sensors, distributed source coding, information bottleneck

## Abstract

This paper addresses the optimization of distributed compression in a sensor network with partial cooperation among sensors. The widely known Chief Executive Officer (CEO) problem, where each sensor has to compress its measurements locally in order to forward them over capacity limited links to a common receiver is extended by allowing sensors to mutually communicate. This extension comes along with modified statistical dependencies among involved random variables compared to the original CEO problem, such that well-known outer and inner bounds do not hold anymore. Three different inter-sensor communication protocols are investigated. The successive broadcast approach allows each sensor to exploit instantaneous side-information of all previously transmitting sensors. As this leads to dimensionality problems for larger networks, a sequential point-to-point communication scheme is considered forwarding instantaneous side-information to only one successor. Thirdly, a two-phase transmission protocol separates the information exchange between sensors and the communication with the common receiver. Inspired by algorithmic solutions for the original CEO problem, the sensors are optimized in a greedy manner. It turns out that partial communication among sensors improves the performance significantly. In particular, the two-phase transmission can reach the performance of a fully cooperative CEO scenario, where each sensor has access to all measurements and the knowledge about all channel conditions. Moreover, exchanging instantaneous side-information increases the robustness against bad Wyner–Ziv coding strategies, which can lead to significant performance losses in the original CEO problem.

## 1. Introduction

This contribution considers a special case of the distributed source coding problem where each sensor observes the same source signal. In order to forward their measurements over capacity limited links to a common receiver, the sensors have to compress their measurements. In the case where a direct communication among operating sensors is not possible, this problem is termed as the CEO problem. Here, the compression at each sensor is optimized according to the Wyner–Ziv coding principle exploiting only statistical side-information. Within this paper, the term CEO problem always stands for this non-cooperative CEO problem, which means that sensors cannot communicate with each other during runtime.

We extend this scenario and allow sensors to cooperate with each other by exchanging instantaneous side-information. The fully cooperative Chief Executive Officer (fcCEO) problem is obtained if sensors can forward their uncompressed observations over inter-sensor links to all other sensors. The partially cooperative Chief Executive Officer (pcCEO) problem represents a scenario where instantaneous side-information is compressed before it is forwarded to other sensors.

### 1.1. The CEO Problem

The CEO problem has been investigated for various system assumptions which mainly differ in the distribution of the variable of interest and the applied distortion measure. The quadratic Gaussian CEO problem considers jointly Gaussian signals and the mean squared error (MSE) distortion measure [1,2,3,4,5,6]. Using an infinite number of encoders, Oohama analytically derived an asymptotic version of the sum-rate distortion in [1]. In [2], the authors investigated the influence of cooperating and non-cooperating encoders on the distortion measure. It turns out that the distortion decreases asymptotically with the reciprocal sum-rate *R* for non-cooperating encoder. In scenarios with cooperating sensors, the MSE distortion decays exponentially with $2^{-2R}$. The non-asymptotic case was first investigated in [3] where the authors derived an upper bound on the sum-rate distortion function. Moreover, they showed that this bound is tight when each encoder has the same measurement SNR. Prabhakaran et al. [4], Oohama [5] and Wagner et al. [6] characterized the complete rate-region for this quadratic Gaussian CEO problem. Results also exist for multivariate Gaussian relevant processes [7,8]. The CEO problem for arbitrary discrete source distributions and the logarithmic loss distortion measure has been analyzed in [9,10,11]. For this scenario, Courtade and Weissman completely characterized the CEO rate-region in [9]. In [10,11], asymptotic analyses for an infinite number of sensors have been performed. Using the Hamming distance as a distortion measure, in [10] an inevitable loss in error rate performance due to non-cooperating sensors has been discovered. For arbitrary distortion measures, a scaling law on the sum-rate distortion function has been derived in [11]. In [12,13], the authors developed a variational bound on the optimal trade-off between relevance and complexity of the CEO setup and used neural networks for encoder and decoder to compute this bound.

There exist several algorithmic approaches to solve the CEO problem [8,14,15,16,17]. In our previous work [14,15], a Greedy Distributed Information Bottleneck (GDIB) algorithm based on the inner bound of the CEO problem with log-loss distortion measure defined in [9] has been introduced to determine extreme points of the contra-polymatroid solution space. Equivalently to Wyner–Ziv coding, the algorithm optimizes the quantizer of a specific sensor using the mappings of previously designed quantizers as statistical side-information. We showed that the GDIB algorithm outperforms an individual scalar information bottleneck optimization of sensors without Wyner–Ziv coding, especially for larger networks. For asymmetric scenarios, we demonstrated the dependency of the performance on the optimization order, i.e., the Wyner–Ziv coding strategy. Since the memory complexity of this optimization algorithm depends exponentially on the network size, we introduced a way to compress the Wyner–Ziv side-information by means of the information bottleneck principle. However, there still remains a large performance gap between non-cooperative and fully cooperative distributed compression.

### 1.2. Partially Cooperating Sensors

In order to close the gap between non-cooperative and fully cooperative distributed compression, we consider an extension of the CEO system model allowing partial cooperation among sensors. While a rich literature can be found on the classical CEO problem, less results are known for partially cooperating sensors. Most work has been done for jointly Gaussian signals because they allow an analytical treatment at least in parts. In [18], it is shown that cooperation among sensors can reduce the compression sum-rate except for the quadratic Gaussian CEO problem. In [19], the authors consider estimation problems under communication constraints and propose coding strategies for tree-structured sensor networks. Exploiting the Wyner–Ziv coding principle, the authors developed solutions for general trees and provide particular results for serial and parallel networks. A two sensor system with a Gaussian source was investigated in [20] for two transmission scenarios: orthogonal but rate-limited links between sensors and the common receiver as well as the Gaussian multiple access channel. It was shown that cooperation between the sensors over rate-limited inter-sensor links leads in both cases to substantial gains in terms of the compression sum-rate. Finally, a simple three node network consisting of encoder, helper and decoder was analyzed in [21]. The authors showed that side-information provided by the helper to the encoder need not exceed that given to the decoder.

### 1.3. Structure and Notation

This paper is structured as follows: Section 2 gives a short introduction to the information bottleneck principle. Section 3 and Section 4 introduce the non-cooperative distributed sensing scenario with the Greedy Distributed Information Bottleneck as an algorithmic solution defined in [14,15] and the fully cooperative distributed sensing scenario being equivalent to a centralized quantization approach, respectively. The main contribution of this paper can be found in Section 5, which introduces the partially cooperative distributed sensing scenario containing three different inter-sensor communication protocols, i.e., successive broadcasting, successive point to point transmission and two-phase transmission. Section 6 concludes this paper.

Throughout this paper, the following notation is used. Calligraphic letters 𝒳,𝒴,Ƶ denote random variables with realizations x,y,z, which are elements of the sets 𝕏,𝕐,ℤ with cardinalities |𝕏|, |𝕐| and |ℤ|, respectively. Bold letters $y={\left[y_{1}\;\ldots \;y_{M}\right]}^{T}$ denote vectors while boldface calligraphic letters 𝓨,Ƶ denote multivariate random variables. Note that ${Ƶ}_{<m}$ covers only the processes ${Ƶ}_{1}$ to ${Ƶ}_{m-1}$. I(𝒳;𝒴) represents the mutual information between the random variables *𝒳* and *𝒴*. Conditional and joint probability mass functions (pmfs) are termed p(y|x) and p(x,y), respectively. The Kullback-Leibler (KL) divergence is given as $D_{KL}[\cdot ∥\cdot ]$. Finally, the expectation of a function f(x) with respect to the random variable *𝒳* is denoted as ${𝔼}_{𝒳}[f(𝒳)]$.

## 2. The Information Bottleneck Principle

The information bottleneck (IB) principle was first introduced by Tishby et al. in [22,23] and defines a clustering framework based on information theoretic measures. An overview about algorithmic solutions for this basic optimization problem is given in [23,24]. The IB principle finds application in various fields in communications [25,26,27,28,29,30].

**Figure 1:**
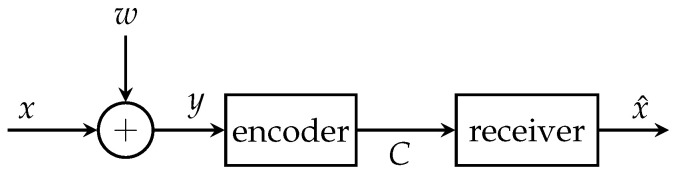
Illustration of a remote sensing problem for a single sensor.

**Figure 2:**
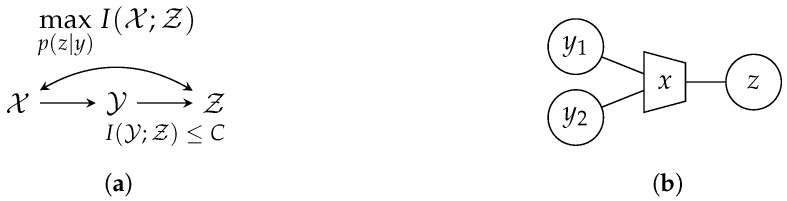
(**a**) Illustration of the IB setup, (**b**) Exemplary IB graph.

The general IB setup is depicted in Figure 1. It contains the relevant process *𝒳*, a noisy observation *𝒴* of *𝒳* and a compressed version *Ƶ* of *𝒴*. The IB approach aims to optimize the mapping p(z|y) in order to preserve as much information about the relevant process *𝒳* in *Ƶ* as possible. More precisely, it tries to maximize the relevant mutual information I(𝒳;Ƶ) while fulfilling a rate constraint I(𝒴;Ƶ)≤C. This general goal is summarized in Figure 2a. The optimization can be formulated as a maximization of the Lagrangian function

(1) $$\begin{array}{cc} L_{\mathrm{IB}}\; & =I(𝒳;Ƶ)-\beta I(𝒴;Ƶ). \end{array}$$

It turns out to be a non-convex optimization problem, since I(𝒳;Ƶ) and I(𝒴;Ƶ) are both convex functions of the mapping p(z|y). The parameter β is a trade-off parameter steering the focus between the preservation of relevant information and the compression of the observation. In the case of β=0, the focus only lies on preservation of relevant information. By increasing β the compression becomes more and more important up to the case of β→∞. Here, the functional in (1) becomes maximal if I(𝒴;Ƶ)=0, which means that all information is compressed to a single cluster. Therefore, the parameter β can be used to adjust the compression rate I(𝒴;Ƶ) in order to fulfill a desired rate constraint I(𝒴;Ƶ)≤C. Since the compression-rate curve is a monotonic increasing function in $\frac{1}{\beta }$, a simple bi-section search can be applied. The optimization problem in (1) can be solved by taking the derivative with respect to the mapping p(z|y) and equating it to zero. It results in the implicit update equation

(2) $$p\left(z|y\right)=\frac{e^{-d_{\beta }\left(y,z\right)}}{\sum_{z}e^{-d_{\beta }\left(y,z\right)}}$$

with

(3) $$\begin{array}{cc} d_{\beta }\left(y,z\right) & =\frac{1}{\beta }D_{KL}[p(x|y)∥p(x|z)]-\log p\left(z\right)\\ \; & =\frac{1}{\beta }{𝔼}_{𝒳|y}[\log\frac{p(x|y)}{p(x|z)}]-\log p\left(z\right). \end{array}$$

In (3), $D_{KL}[p(x|y)∥p(x|z)]$ denotes the Kullback–Leibler divergence. This implicit solution can be solved by an iterative Blahut–Arimoto-like algorithm.

In the case of focusing solely on preservation of relevant information with β=0, the optimization algorithm yields a deterministic clustering p(z|y)∈{0,1}. For β>0, the clustering p(z|y)∈[0,1] is generally stochastic. The IB method can easily be extended to multiple input values. A graphical tool for visualization are IB graphs [31]. Figure 2b illustrates an example where the observations $y_{1},y_{2}$ are compressed into the cluster index *z*. The trapezoid represents the IB compression with respect to the relevant variable written inside the trapezoid.

## 3. Non-Cooperative Distributed Sensing System

**Figure 3:**
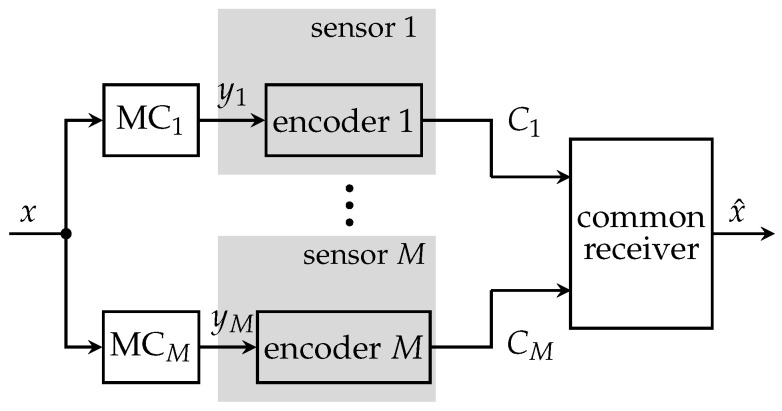
Non-cooperative distributed sensing system with *M* sensors, a common receiver and individual link capacities $C_{m}$.

Figure 3 illustrates the CEO system model without communication among operating sensors. Here, *M* sensors observe noisy versions $y_{m}$ of the same relevant signal *x*. The measurement processes can be modeled as statistically independent memoryless channels (MCs).

Exemplarily, a measurement $y_{m}=x+w_{m}$ represents the relevant signal *x* corrupted by zero mean white Gaussian measurement noise $w_{m}$ with measurement signal-to-noise-ratio (SNR) $\gamma_{m}=\frac{\sigma_{x}^{2}}{\sigma_{w_{m}}^{2}}$, where $\sigma_{x}^{2}$, $\sigma_{w_{m}}^{2}$ denote signal and noise variances, respectively. In order to be able to forward the measurements over capacity limited links with capacities $C_{1},\ldots ,C_{M}$, each sensor has to compress its observations using a specific encoding process. More precisely, each sensor compresses its observations $y_{m}$ to a cluster index $z_{m}$ using the mapping $p(z_{m}|y_{m})$ leading to the Markov property:

(4) $$\begin{array}{c} p\left(z,y,x\right)=\prod_{m=1}^{M}p\left(z_{m}|y_{m}\right)p\left(y_{m}|x\right)p\left(x\right). \end{array}$$

The encoding process contains a second lossy compression step if the mapping $p(z_{m}|y_{m})$ is stochastic and lossless entropy coding if the mapping $p(z_{m}|y_{m})$ is deterministic. Therefore, a compressed version of the index $z_{m}$ is transmitted without any further loss to the common receiver. The optimization of $p(z_{m}|y_{m})$ for each sensor is done offline.

The mathematical analysis of the CEO problem and the structure of its rate-region for discrete input alphabets and the log-loss distortion measure was presented in [9] and exploits (4). It was proved that the extreme points of the contra-polymatroid solution space can be determined by greedy algorithms as the one described next. Since the communication among sensors during run-time is not possible in this approach, the solution represents a lower bound on the performance of cooperative distributed compression in this paper.

An algorithmic solution to solve the CEO problem has previously been proposed in [14,15] as the so called Greedy Distributed Information Bottleneck (GDIB) algorithm. It is based on the inner bound of the CEO rate-region for the logarithmic loss distortion measure [9] and optimizes the quantization at the sensors successively. Replacing the logarithmic loss function H(𝒳|Ƶ) by the relevant mutual information I(𝒳;Ƶ) delivers the optimization problem

(5) $$\begin{array}{c} \max_{P}I\left(𝒳;Ƶ\right)\;s.t.\;I\left({𝓨}_{𝕊};{Ƶ}_{𝕊}|{Ƶ}_{\bar{𝕊}}\right)\leq \sum_{m\in 𝕊}C_{m}\\ \forall \;𝕊\subseteq \{1,2,\ldots ,M\}. \end{array}$$

The set $P=\;\left[\begin{array}{ccc} p(z_{1}|y_{1}) & \cdots & p(z_{M}|y_{M}) \end{array}\right]$ defines the set of all mappings. According to [9], the compression rates $I({𝓨}_{𝕊};{Ƶ}_{𝕊}|{Ƶ}_{\bar{𝕊}})$ are supermodular set functions with respect to the sets 𝕊 [32], while the relevant information I(𝒳;Ƶ) does not depend on 𝕊. Therefore, the greedy optimization structure of the GDIB algorithm is optimal and finds the extreme points of the solution space. It has to be emphasized that since the GDIB algorithm is based on the inner bound of the rate-region, it does not find the complete rate-region of the CEO problem. Following this approach, *M* IB related Lagrangian optimization problems are obtained, one for each sensor.

(6) $$\begin{array}{cc} L_{\mathrm{GDIB}}^{\left(1\right)} & =I\left(𝒳;{Ƶ}_{1}\right)-\beta_{1}I\left({𝒴}_{1};{Ƶ}_{1}\right)\\ \; & \vdots \end{array}$$

(7) $$\begin{array}{cc} L_{\mathrm{GDIB}}^{\left(M\right)} & =I\left(𝒳;{Ƶ}_{M}|{Ƶ}_{<M}\right)-\beta_{M}I\left({𝒴}_{M};{Ƶ}_{M}|{Ƶ}_{<M}\right) \end{array}$$

Obviously, the optimization problem of the first sensor resembles the optimization problem for the scalar IB problem given in (1) since there is no predecessor. Subsequent sensors exploit the mappings of previously designed quantizers as statistical side-information leading to the well-known Wyner–Ziv coding strategy. Naturally, each Lagrange multiplier $\beta_{m}$ has to be chosen such that the corresponding compression rate fulfills the individual rate constraint $I\left({𝒴}_{m};{Ƶ}_{m}|{Ƶ}_{<m}\right)\leq C_{m}$. The objectives in (6) and (7) can be solved by equating the derivative with respect to the mapping $p(z_{m}|y_{m})$ to zero delivering the update rule

(8) $$\begin{array}{c} p\left(z_{m}|y_{m}\right)=\frac{e^{-d_{\beta_{m}}\left(y_{m},z_{m}\right)}}{\sum_{z_{m}}e^{-d_{\beta_{m}}\left(y_{m},z_{m}\right)}} \end{array}$$

with the exponent

(9) $$\begin{array}{cc} d_{\beta_{m}}\left(y_{m},z_{m}\right)≔E_{{Ƶ}_{<m}|y_{m}} & \left[\frac{1}{\beta_{m}}D_{KL}[p\left(x|y_{m},z_{<m}\right)∥p\left(x|z_{\leq m}\right)]-\log p\left(z_{m}|z_{<m}\right)\right]. \end{array}$$

Similar to the scalar IB optimization, the implicit expression in (8) can be solved using a Blahut–Arimoto like algorithm, providing local optimal solutions. It has to be mentioned that for asymmetric scenarios, this optimization has to be performed for all M! possible permutations of the optimization order to find the best solution.

A detailed derivation and performance analysis of this algorithm can be found in [14,15]. If the capacity is equally distributed over all sensors in the network, e.g., sensors share the same channel in an orthogonal way and a round robin fashion, numerical results demonstrate that the GDIB algorithm outperforms an individual scalar IB optimization at each sensor. However, there is still a large gap to the performance of a fcCEO scenario, which is defined in Section 4. Moreover, in asymmetric scenarios, the performance highly depends on the optimization order. Although no clear conclusion about the optimal Wyner–Ziv coding strategy can be drawn, a good solution can be expected when starting the optimization with the best forward channel conditions, i.e., the lowest compression (highest compression rate).

## 4. Fully Cooperative Distributed Sensing—A Centralized Quantization Approach

**Figure 4:**
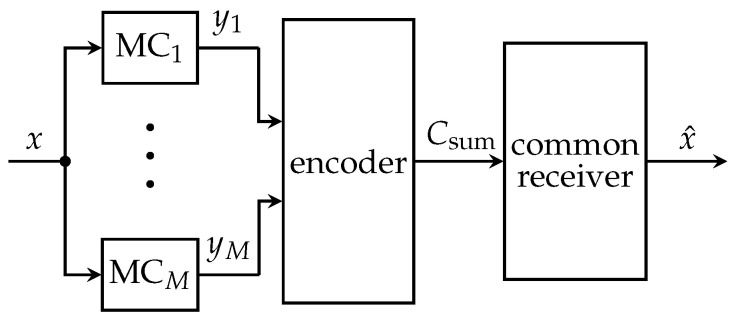
Model of a centralized compression approach representing the fully cooperative Chief Executive Officer scenario.

This section introduces the fcCEO scenario, which considers distributed sensors being able to forward their uncompressed observations to all other sensors in the network over ideal noiseless inter-sensor links. In this case, sensors can perfectly exchange their measurements $y_{m}$ before they jointly compress the received signals taking into account the rate constraints of all individual forward channels. Naturally, the exchange has to be done by a two-phase transmission protocol, consisting of a cooperation phase and a transmission phase. During the cooperation phase sensors exchange information until every sensor knows measurements $y={\left[y_{1}\ldots y_{M}\right]}^{T}$ of all *M* sensors. The actual forwarding of the compressed observations to the common receiver is performed during the transmission phase. This full cooperation is equivalent to a single central quantizer having access to all measurements **y** as depicted in Figure 4. Applying the IB principle, this central quantizer can be designed in order to compress the vector **y** onto a cluster index *z* using the mapping p(z|y), which motivates the name centralized IB (CIB) for the algorithmic solution in a fcCEO scenario. The optimization problem can be formulated as the maximization of

(10) $$\begin{array}{c} L_{\mathrm{CIB}}=I\left(𝒳;Ƶ\right)-\beta I\left(𝓨;Ƶ\right) \end{array}$$

and is solved using update Equation (2) with (3) substituting the scalar *y* by vector **y**. The number of output clusters |ℤ| has to be chosen to $\left|\mathbb{Z}\right|=\prod_{m=1}^{M}\left|{\mathbb{Z}}_{m}\right|$ while the single link from the imaginary central quantizer to the receiver in Figure 4 has a channel capacity of $C_{\mathrm{sum}}=\sum_{m=1}^{M}C_{m}$. The actual transmission over the *M* links has to be coordinated such that each sensor *m* transmits a specific part of the bits corresponding to its link capacity $C_{m}$.

In the special case of the measurement process being modeled as additive noise, the algorithm can be simplified to a scalar optimization problem where maximum ratio combining of all inputs $y_{m}$ delivers a scalar sufficient statistics

$$\bar{y}=\sum_{m=1}^{M}\gamma_{m}\cdot y_{m}$$

of the desired relevant signal *x* with an overall SNR $\gamma =\sum_{m}\gamma_{m}$. The solution of the fcCEO scenario serves as an upper bound in this paper.

## 5. Partially Cooperative Distributed Sensing

In order to investigate how the gap between non-cooperative and fully-cooperative distributed compression can be reduced, partially cooperating sensors shall now be considered. Partial cooperation means a limited exchange of instantaneous side-information among the sensors during runtime due to a rate-limitation of inter-sensor links. Non-cooperative CEO and fully-cooperative CEO problems represent the extreme cases for zero rate and unlimited rate inter-sensor links, respectively. The rate limitation requires the compression of instantaneous side-information before forwarding it to other sensors.

In this paper, only deterministic mappings are considered for this compression, while indexes $z_{m}$ are still obtained by stochastic mappings. This is motivated by the fact that deterministic mappings do not require further lossy compression and the resulting side-information indices $s_{m}$ can be exploited at other sensors by choosing a particular mapping $p(z_{m}|y_{m})$ from a list of possible mappings designed offline in advance. As a consequence, the compression rates for instantaneous side-information can only be adjusted by changing the cardinalities $|{𝕊}_{m}|$. For all results presented below, inter-sensor links are modeled as bit pipes being able to deliver $s_{m}$ reliably.

The GDIB algorithm to solve the non-cooperative CEO problem is based on the inner bound (5) of the CEO rate-region. Moreover, a greedy optimization approach is optimal due to the supermodularity of the compression rates in (5). Both require the Markovian structure in (4). However, cooperation among sensors changes the Markovian structure and implies different statistical dependencies among involved random variables. As (4) does not hold anymore in pcCEO scenarios, the inner bound on the rate-region in (5) cannot be utilized to find solutions of the pcCEO scenario. To the knowledge of the authors, tight bounds on the rate-region are not available for the cooperative case. Therefore, a heuristic approach based on the greedy optimization structure of the GDIB algorithm will be applied to solve the pcCEO scenario, which is not proven to be optimal. Nevertheless, the numerical evaluation of the found solutions demonstrate their usefulness. However, the computation of required pmfs becomes more challenging and results in recursive calculations given in Appendix A.1 and Appendix A.2 because the Markovian structure of (4) does not hold anymore in pcCEO scenarios.

This paper introduces three different inter-sensor communication protocols for exchanging this instantaneous side-information: successive broadcasting, a successive point-to-point transmission and a two-phase transmission. The first two protocols perform the exchange of instantaneous side-information $s_{m}$ with other sensors and the forwarding of compressed versions of $z_{m}$ to the common receiver in the same time slot. Contrarily, the two-phase transmission protocol separates the exchange of instantaneous side-information among sensors and the communication with the common receiver into two distinct phases. The latter starts after the exchange among sensors has been completed such that all sensors have (approximately) the same amount of side-information.

### 5.1. Successive Broadcasting Protocol

**Figure 5:**
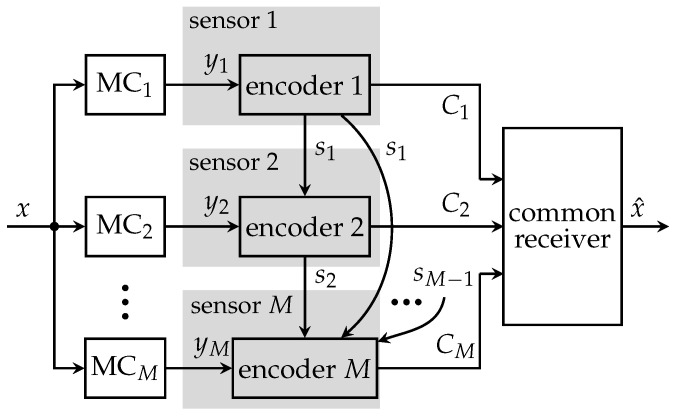
Partially cooperative CEO scenario using broadcast exchange of side-information among sensors.

The system model for the successive broadcasting protocol is illustrated in Figure 5. In the same time slot, sensor m−1 not only forwards a compressed version of the quantization index $z_{m-1}$ to the common receiver, but also broadcasts instantaneous side-information $s_{m-1}$ to all other sensors. However, due to the greedy optimization structure, only subsequent sensors can exploit this instantaneous side-information. Thus, sensor *m* can exploit indices $s_{<m}$ of all previously transmitting sensors in order to select its quantization index $z_{m}$ as well as a new instantaneous side-information index $s_{m}$. This scenario leads to the Markov model

(11) $$\begin{array}{cc} p(x,y,z,s)\; & =\prod_{m=1}^{M}p\left(z_{m}|y_{m},s_{<m}\right)p\left(s_{m}|y_{m},s_{<m}\right)p\left(y_{m}|x\right)p\left(x\right). \end{array}$$

**Figure 6:**
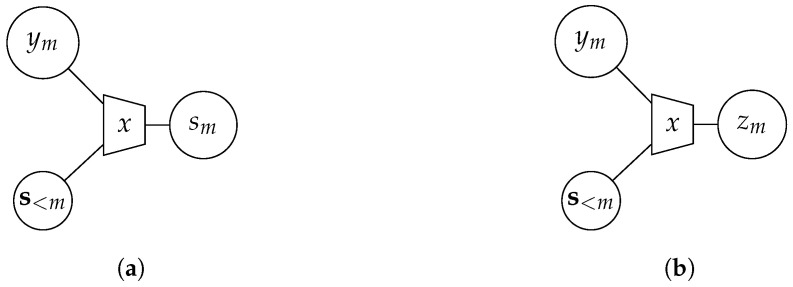
Graphical illustration of IB fusion of involved inputs to determine instantaneous side-information $s_{m}$ (**a**) and the quantizer sensor output $z_{m}$ (**b**) for a broadcast exchange of instantaneous side-information.

The indices $s_{m}$ and $z_{m}$ are obtained by deterministic mappings $p\left(s_{m}|y_{m},s_{<m}\right)\in \left\{0,1\right\}$ and stochastic mappings $p\left(z_{m}|y_{m},s_{<m}\right)\in \left[0,1\right]$, respectively. The design of these mappings can be performed offline leveraging the IB principle as illustrated in Figure 6. It combines the observation $y_{m}$ and the instantaneous side-information $s_{<m}$ to indexes $s_{m}$ and $z_{m}$ while maintaining as much information as possible about the relevant signal *x*.

#### 5.1.1. Generation of Broadcast Side-Information

The design of $p(s_{m}|y_{m},s_{<m})$ is inspired by the general GDIB algorithm, i.e., the optimization is done in a greedy manner. Again, there emerges one optimization problem for each sensor:

(12) $$\begin{array}{cc} L_{\mathrm{BC}-\mathrm{SIDE}}^{\left(1\right)} & =I\left(𝒳;{𝒮}_{1}\right)-\beta I\left({𝒴}_{1};{𝒮}_{1}\right)\\ \; & \vdots \end{array}$$

(13) $$\begin{array}{cc} L_{\mathrm{BC}-\mathrm{SIDE}}^{\left(M-1\right)} & =I\left(𝒳;{𝒮}_{M-1}|{𝓢}_{<M-1}\right)-\beta I\left({𝒴}_{M-1};{𝒮}_{M-1}|{𝓢}_{<M-1}\right). \end{array}$$

The optimization problem of the first sensor equals the individual scalar optimization without any side-information at all, as described in Section 2. Subsequent sensors combine the instantaneous side-information of all previously transmitting sensors $s_{<m}$ with its observation $y_{m}$. The relevant mutual information and the compression rate of sensor *m* are conditioned on ${𝓢}_{<m}$ since broadcasting instantaneous side-information ensures all successive sensors to have access to $s_{<m}$ allowing Wyner–Ziv coding for generating $s_{m}$. Each optimization problem given in (12) and (13) can be solved by taking the derivative with respect to the mapping $p(s_{m}|y_{m},s_{<m})$ and equating it to zero. This results in the implicit update equation

(14) $$p\left(s_{m}|y_{m},s_{<m}\right)=\frac{e^{-d_{\beta_{m}}\left(y_{m},s_{m},s_{<m}\right)}}{\sum_{s_{m}}e^{-d_{\beta_{m}}\left(y_{m},s_{m},s_{<m}\right)}}$$

with

(15) $$\begin{array}{c} d_{\beta_{m}}\left(y_{m},s_{m},s_{<m}\right)≔\frac{1}{\beta_{m}}D_{KL}\left[p\left(x|y_{m},s_{<m}\right)∥p\left(x|s_{\leq m}\right)\right]-\log p\left(s_{m}|s_{<m}\right). \end{array}$$

As in the general GDIB algorithm, the implicit update equation in (14) can be solved using a Blahut–Arimoto like algorithm resulting in local optimal solutions.

##### Algorithmic pcCEO Solution for the Successive Broadcasting Protocol

After designing the mapping for the instantaneous side-information, the mapping $p(z_{m}|y_{m},s_{<m})$ can be optimized, again by means of the IB principle. Therefore, the original GDIB algorithm is modified to exploit the broadcasted instantaneous side-information, defining the GDIB-BC algorithm. The optimization problem for each sensor is given as

(16) $$\begin{array}{cc} L_{\mathrm{GDIB}-\mathrm{BC}}^{\left(1\right)} & =I\left(𝒳;{Ƶ}_{1}\right)-\beta_{1}I\left({𝒴}_{1};{Ƶ}_{1}\right)\\ \; & \vdots \end{array}$$

(17) $$\begin{array}{cc} L_{\mathrm{GDIB}-\mathrm{BC}}^{\left(M\right)} & =I\left(𝒳;{Ƶ}_{M}|{Ƶ}_{<M}\right)-\beta_{M}I\left({𝒴}_{M},{𝓢}_{<M};{Ƶ}_{M}|{Ƶ}_{<M}\right). \end{array}$$

The main difference to the original GDIB optimization problem in (6) and (7) lies in the definition of the compression rate $I({𝒴}_{m},{𝓢}_{<m};{Ƶ}_{m}|{Ƶ}_{<m})$ which emerges from the combination of the observation $y_{m}$ and the instantaneous side-information $s_{<m}$. Taking the derivative of the optimization problem for sensor *m* with respect to the mapping $p(z_{m}|y_{m},s_{<m})$ and equating it to zero delivers

(18) $$p\left(z_{m}|y_{m},s_{<m}\right)=\frac{e^{-d_{\beta_{m}}\left(y_{m},z_{m},s_{<m}\right)}}{\sum_{z_{m}}e^{-d_{\beta_{m}}\left(y_{m},z_{m},s_{<m}\right)}}$$

with

(19) $$d_{\beta_{m}}\left(y_{m},z_{m},s_{<m}\right)≔E_{{Ƶ}_{<m}|y_{m},s_{<m}}[\frac{1}{\beta_{m}}\cdot \;D_{KL}[p\left(x|y_{m},s_{<m},z_{<m}\right)∥p\left(x|z_{\leq m}\right)]-\log p\left(z_{m}|z_{<m}\right)].$$

Again, the implicit update equation in (18) can be solved using a Blahut–Arimoto like algorithm. The extended Blahut–Arimoto like algorithm to design the mapping $p(z_{m}|y_{m},s_{<m})$ of sensor *m* for a specific Lagrange parameter $\beta_{m}$ and instantaneous side-information $s_{<m}$ is given in Algorithm 1. The input pmf $p(y_{m-1},s_{<m-1},z_{<m-1},x)$ can be computed during the optimization of previous sensors. Lines 3 to 5 determine the required pmfs for the calculation of the KL-divergence of (19) in lines 6 to 9. The statistical distance of (19) is determined in lines 10 to 14. It is used to update the quantizer mapping $p(z_{m}|y_{m},s_{<m})$ of sensor *m*. This procedure is repeated until no significant changes of the desired mappings occur anymore. The algorithm returns the updated mapping $p(z_{m}|y_{m},s_{<m})$ as well as the pmf $p(y_{m},s_{<m},z_{<m},x)$, which is used as an input for the successive sensor.
**Algorithm 1:** Extended Blahut–Arimoto algorithm for broadcast cooperating sensors.
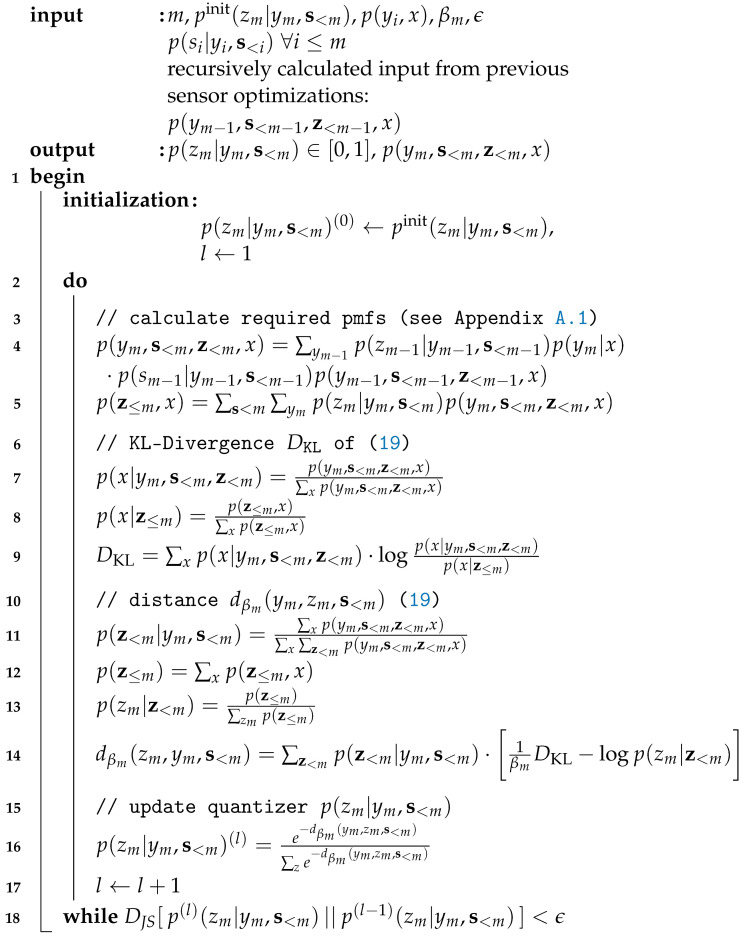


The parameter $\beta_{m}$, which determines the compression rate at sensor *m*, has to be adjusted such that $I\left({𝒴}_{m},{𝓢}_{<m};{Ƶ}_{m}|{Ƶ}_{<m}\right)\leq C_{m}$ is fulfilled. Similar to the original GDIB algorithm, the GDIB-BC algorithm has to be performed for each sensor and all possible optimization orders.

#### 5.1.2. Evolution of Instantaneous Side-Information

**Figure 7:**
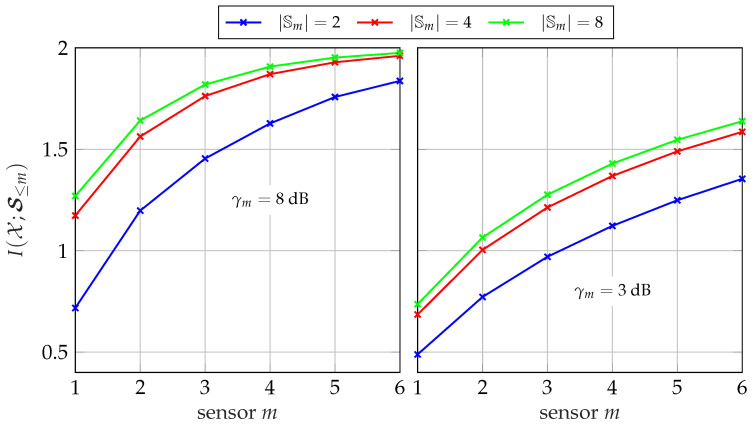
Available mutual information $I(𝒳;{𝓢}_{\leq m})$ for sensor *m* in a network with M=6 sensors and different cardinalities $|{𝕊}_{m}|$ using the successive broadcasting protocol; |𝕏|=4, $|{𝕐}_{m}|\;=64$.

Figure 7 illustrates the amount of instantaneous side-information available at the different sensors in a network of size M=6 considering the broadcast of side-information. It depicts the relevant mutual information $I(𝒳;{𝓢}_{\leq m})$ versus the sensor number *m* for different cardinalities $|{𝕊}_{m}|$ and SNRs $\gamma_{m}$. The relevant signal is chosen to be a uniformly distributed 4-ASK signal leading to |𝕏|=4. As expected, the amount of available instantaneous side-information increases with each additional sensor for all $|{𝕊}_{m}|$ and $\gamma_{m}$. To be more specific, the resolution and the quality of instantaneous side-information available at sensor *m* increases with growing *m*. In the considered symmetric scenario, the amount of information $I(𝒳;{𝒮}_{m}|{𝓢}_{<m})$ a sensor can contribute to $I(𝒳;{𝓢}_{\leq m})$ gets smaller for each additional sensor and the slopes of the curves decrease. Since one bit is not enough to represent the information of |𝕏|=4, the largest gain can be observed between $|{𝕊}_{m}|\;=2$ and $|{𝕊}_{m}|\;=4$. Increasing the cardinality further to $|{𝕊}_{m}|\;=8$ results only in a small additional improvement. Certainly, this observation depends on the relevant signal *𝒳* and can not be generalized.

#### 5.1.3. Performance for Different Network Sizes

**Figure 8:**
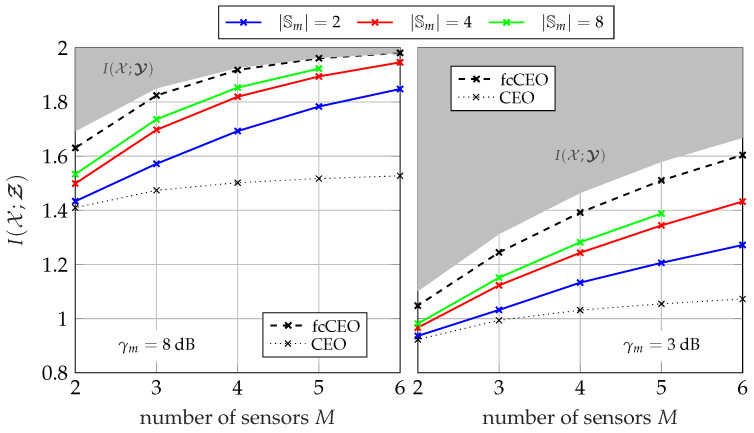
Relevant mutual information I(𝒳;Ƶ) versus the network size for a fixed sum-rate of $C_{\mathrm{sum}}=2.5$ bit/s/Hz and $C_{m}=\frac{C_{\mathrm{sum}}}{M}$ using the successive broadcasting protocol with different cardinalities $|{𝕊}_{m}|$; |𝕏|=4, $|{𝕐}_{m}|\;=64$, $|{\mathbb{Z}}_{m}|\;=4$.

**Figure 9:**
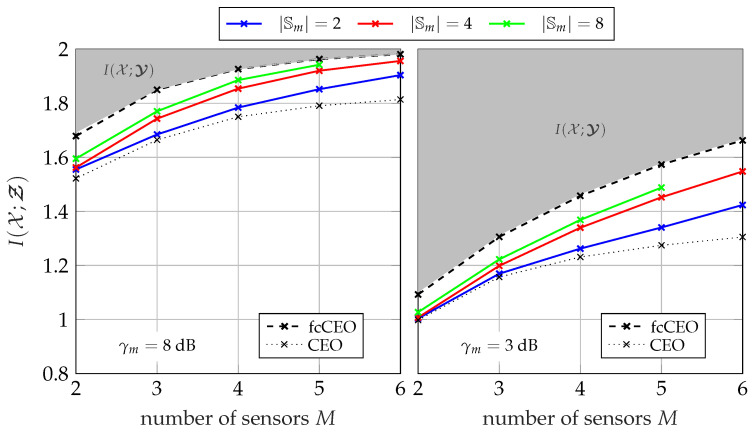
Relevant mutual information I(𝒳;Ƶ) versus the network size for a fixed sum-rate of $C_{\mathrm{sum}}=4$ bit/s/Hz and $C_{m}=\frac{C_{\mathrm{sum}}}{M}$ using the successive broadcasting protocol with different cardinalities $|{𝕊}_{m}|$; |𝕏|=4, $|{𝕐}_{m}|\;=64$, $|{\mathbb{Z}}_{m}|\;=4$.

Figure 8 and Figure 9 illustrate the overall performance of the GDIB-BC approach when broadcasting instantaneous side-information for different network sizes. The relevant mutual information I(𝒳;Ƶ) is depicted versus the number of sensors *M* in the network. The gray colored area represents the non-achievable region, since I(𝒳;Ƶ) cannot exceed I(𝒳;𝓨) due to the data-processing inequality. Both figures consider a scenario where all sensors in the network share the same channel to the common receiver with a fixed sum-rate $C_{\mathrm{sum}}$ in an orthogonal way and a round robin fashion. Consequently, larger network sizes correspond to smaller individual capacities $C_{m}=\frac{C_{\mathrm{sum}}}{M}$ for each forward link. The performance of partially cooperating sensors broadcasting instantaneous side-information (pcCEO-BC) is compared to the non-cooperative case (CEO) of Section 3 and the fully cooperative case (fcCEO) of Section 4. As already mentioned, these two scenarios provide upper and lower bounds. In general, it can be observed that increasing the number of sensors in the network also increases the overall relevant mutual information I(𝒳;Ƶ). This holds even for the case without cooperation, since each sensor applies Wyner–Ziv coding and exploits the mapping of previously designed quantizers as statistical side-information [14]. Independent of the cardinality $|{𝕊}_{m}|$, the performance of the pcCEO-BC scenario is superior to the case without cooperation among sensors. This difference grows for larger network sizes because the amount of information $s_{<m}$ has about the relevant variable *x* increases. As expected from Figure 7, increasing the cardinality $|{𝕊}_{m}|$ not only improves the relevant information $I(𝒳;{𝓢}_{\leq m})$, but also the overall performance measured by I(𝒳;Ƶ). However, it can be observed that even for large $|{𝕊}_{m}|$ there remains a gap to the fcCEO upper bound, especially for smaller network sizes or lower SNRs. This gap can be explained by the successive transmission protocol resulting in a gradually increasing amount of instantaneous side-information at the sensors. For instance, the first sensors does not profit at all from the partial cooperation in contrast to the fcCEO scenario where all sensors exploit almost the same amount of side-information.

Considering the pmfs in Algorithm 1, it becomes obvious that larger networks might suffer from the curse of dimensionality. More precisely, pmfs like $p(y_{m},s_{<m},z_{<m},x)$ can become very large during the optimization for larger network sizes. Moreover, the mapping $p(z_{m}|y_{m},s_{<m})$ also depends on the network size, i.e., this problem does not only occur during the optimization, but also when storing the already optimized mapping. This numerical issue is the reason why there is no result for $|{𝕊}_{m}|\;=8$ and a network size of M=6 in Figure 8 and Figure 9. In this case, it requires 2024 GiB (1 GiB = 1024 MiB, 1 MiB = 1024 KiB, 1 KiB = 1024 byte) just for storing a single instance of the pmf $p(y_{m},s_{<m},z_{<m},x)$.

### 5.2. Successive Point-to-Point Protocol

**Figure 10:**
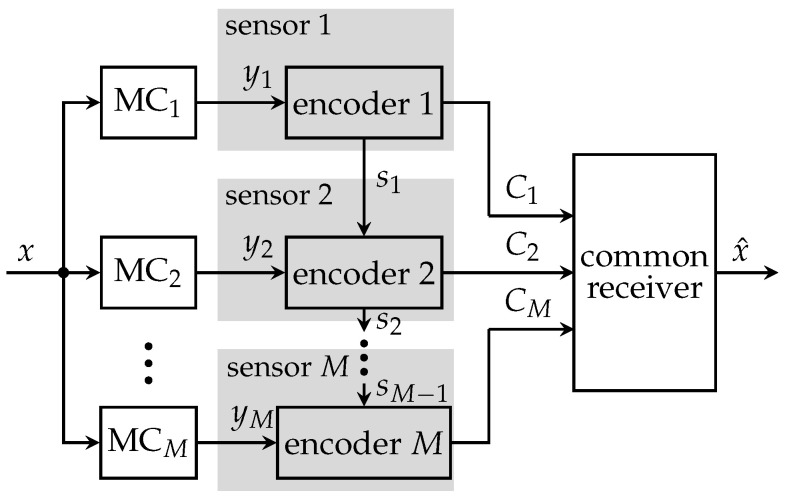
Partially cooperative CEO scenario using successive point-to-point transmission of side-information.

For larger network sizes, broadcasting side-information might not be feasible anymore, since the dimensions of the mappings $p(z_{m}|y_{m},s_{<m})$ and $p(s_{m}|y_{m},s_{<m})$ as well as intermediate pmfs used within the optimization become huge. In order to relax this curse of dimensionality, the successive way of cooperation is exploited and the instantaneous side-information of sensor *m* shall only be forwarded to the direct successor m+1 as depicted in Figure 10. Hence, a sequential chain is established from the first to the last sensor leading to the Markov Model:

(20) $$\begin{array}{c} p\left(x,y,z,s\right)=\prod_{m=1}^{M}p\left(z_{m}|y_{m},s_{m-1}\right)p\left(s_{m}|y_{m},s_{m-1}\right)p\left(y_{m}|x\right)p\left(x\right). \end{array}$$

**Figure 11:**
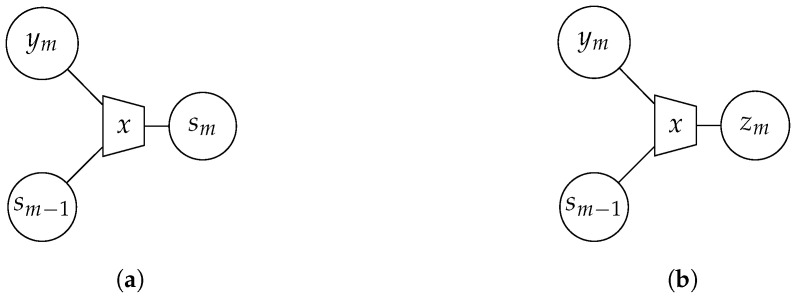
Graphical illustration of IB fusion of two inputs to determine instantaneous side-information $s_{m}$ (**a**) and the quantizer sensor output $z_{m}$ (**b**) for a successive point-to-point transmission of instantaneous side-information.

Again, the instantaneous side-information is obtained by a deterministic mapping optimized by means of the information bottleneck principle, illustrated in Figure 11. With each step in the sequential chain, the information $s_{m}$ has about the relevant signal *x* increases.

#### 5.2.1. Generation of Point-to-Point Side-Information

Similar to the broadcast case, the design of $p(s_{m}|y_{m},s_{m-1})$ is inspired by the original GDIB algorithm. The optimization problem can be formulated in a greedy manner as

(21) $$\begin{array}{cc} L_{\mathrm{PTP}-\mathrm{SIDE}}^{\left(1\right)} & =I\left(𝒳;{𝒮}_{1}\right)-\beta I\left({𝒴}_{1};{𝒮}_{1}\right)\\ \; & \vdots \end{array}$$

(22) $$\begin{array}{cc} L_{\mathrm{PTP}-\mathrm{SIDE}}^{\left(M-1\right)} & =I\left(𝒳;{𝒮}_{M-1}\right)-\beta I\left({𝒴}_{M-1},{𝒮}_{M-2};{𝒮}_{M-1}\right)\;, \end{array}$$

where Equation (21) equals the individual scalar optimization without any side-information. Subsequent sensors combine the instantaneous side-information $s_{m-1}$ sent by the previous sensor with its observation $y_{m}$. In contrast to the broadcast case, the relevant mutual information is not conditioned on ${𝓢}_{<m}$ as in (12) and (13) because sensor *m* will only have access to $s_{m-1}$ and not to indices of any other sensor. Therefore, Wyner–Ziv coding cannot be applied for exchanging instantaneous side-information with the successive point-to-point protocol. The optimization problems can be solved by taking the derivative with respect to the mapping $p(s_{m}|y_{m},s_{m-1})$ and equating it to zero, resulting in the implicit update equation

(23) $$\begin{array}{c} p\left(s_{m}|y_{m},s_{m-1}\right)=\frac{e^{-d_{\beta_{m}}\left(y_{m},s_{m},s_{m-1}\right)}}{\sum_{s_{m}}e^{-d_{\beta_{m}}\left(y_{m},s_{m},s_{m-1}\right)}} \end{array}$$

with

(24) $$\begin{array}{c} d_{\beta_{m}}\left(y_{m},s_{m},s_{m-1}\right)≔\frac{1}{\beta_{m}}\cdot D_{KL}\left[p\left(x|y_{m},s_{m-1}\right)∥p\left(x|s_{m}\right)\right]-\log p\left(s_{m}\right). \end{array}$$

As in the broadcast case, using a Blahut–Arimoto like algorithm to solve the update Equation (23) results in local optimal solutions.

#### 5.2.2. Algorithmic pcCEO Solution Applying the Successive Point-to-Point Protocol

After the optimization of the mapping for instantaneous side-information, the mapping $p(z_{m}|y_{m},s_{m-1})$ can be designed by means of the information bottleneck principle. Inspired by the original GDIB algorithm, the optimization problem can be formulated as

(25) $$\begin{array}{cc} L_{\mathrm{GDIB}-\mathrm{PTP}}^{\left(1\right)} & =I\left(𝒳;{Ƶ}_{1}\right)-\beta_{1}I\left({𝒴}_{1};{Ƶ}_{1}\right)\\ \; & \vdots \end{array}$$

(26) $$\begin{array}{cc} L_{\mathrm{GDIB}-\mathrm{PTP}}^{\left(M\right)} & =I\left(𝒳;{Ƶ}_{M}|{Ƶ}_{<M}\right)-\beta_{M}I\left({𝒴}_{M},{𝒮}_{M-1};{Ƶ}_{M}|{Ƶ}_{<M}\right). \end{array}$$

The main difference to the original GDIB optimization problem in (6) and (7) now lies in the compression rate $I({𝒴}_{m},{𝒮}_{m-1};{Ƶ}_{m}|{Ƶ}_{<m})$, which emerges from the combination of the instantaneous side-information $s_{m-1}$ of the previous sensor m−1 and the observation $y_{m}$ of sensor *m*. Taking the derivative with respect to the mapping $p(z_{m}|y_{m},s_{m-1})$ and equating it to zero, the optimization problem for sensor *m* can be solved, leading to the implicit update equation

(27) $$\begin{array}{c} p\left(z_{m}|y_{m},s_{m-1}\right)=\frac{e^{-d_{\beta_{m}}\left(y_{m},z_{m},s_{m-1}\right)}}{\sum_{z_{m}}e^{-d_{\beta_{m}}\left(y_{m},z_{m},s_{m-1}\right)}} \end{array}$$

with

(28) $$\begin{array}{cc} \; & \;\;d_{\beta_{m}}\left(y_{m},z_{m},s_{m-1}\right)≔E_{{Ƶ}_{<m}|y_{m},s_{m-1}}[\frac{1}{\beta_{m}}\cdot \\ & D_{KL}[p\left(x|y_{m},s_{m-1},z_{<m}\right)∥p\left(x|z_{\leq m}\right)]-\log p\left(z_{m}|z_{<m}\right)]. \end{array}$$

Thus, the mapping $p(z_{m}|y_{m},s_{m-1})$ can be optimized using a Blahut–Arimoto-like algorithm. The specific algorithm for a given sensor *m* and a Lagrange parameter $\beta_{m}$ is given in Algorithm 2. The input pmfs $p(z_{i}|y_{i},s_{i-1})\;\forall i<m$ and $p(s_{i}|z_{\leq i},x)\;\forall i<m$ as well as $p(z_{<m-1},x)$ are calculated in advance by previous sensor optimizations. Lines 3 to 7 calculates required pmfs as given the Appendix A.2. The KL-divergence is calculated in lines 8 to 11. Using this, the statistical distance $d_{\beta_{m}}\left(z_{m},y_{m},s_{m-1}\right)$ of (28) can be calculated in lines 12 to 16, which is then used to update the quantizer mapping $p(z_{m}|y_{m},s_{m-1})$. The algorithm stops if this mapping does not change significantly anymore during subsequent iterations. Finally, the output pmfs $p(s_{m}|z_{\leq m},x)$ and $p(z_{<m},x)$ need to be calculated in lines 21 to 25 for their usage in the optimization of the next sensor.

Similar to the original GDIB algorithm, the optimization needs to be done for all possible optimization orders. A simple bisection search can be applied to find the rate-fulfilling parameter $\beta_{m}$, such that $I\left({𝒴}_{m},{𝒮}_{m-1};{Ƶ}_{m}|{Ƶ}_{<m}\right)\leq C_{m}$ holds.

#### 5.2.3. Evolution of Instantaneous Side-Information

**Figure 12:**
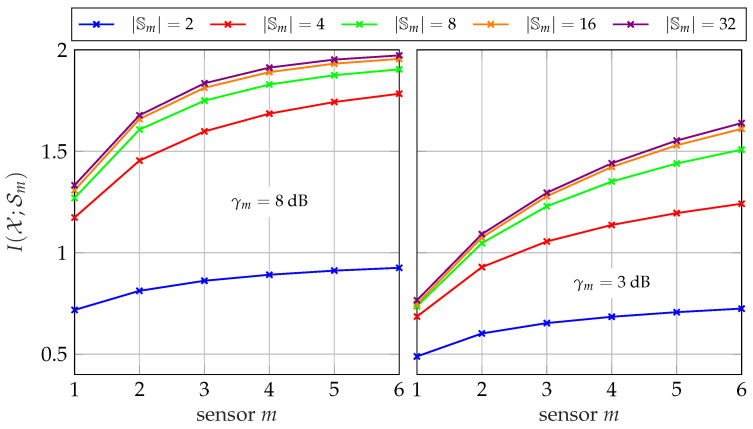
Evolution of $I(𝒳;{𝒮}_{m})$ for sensor *m* in a network with M=6 sensors and different cardinalities $|{𝕊}_{m}|$ for the successive point-to-point transmission protocol; |𝕏|=4, $|{𝕐}_{m}|\;=64$.

Figure 12 illustrates the amount of instantaneous side-information $I(𝒳;{𝒮}_{m})$ at a specific sensor *m* in a network of size M=6 using the successive point-to-point transmission protocol for different cardinalities $|{𝕊}_{m}|$. Obviously, $I(𝒳;{𝒮}_{m})$ increases with each further sensor. The main difference to the broadcast case is that the instantaneous side-information provided to sensor *m* is represented by a single highly compressed index $s_{m-1}$ with cardinality $|{𝕊}_{m-1}|$. While the resolution $|{𝕊}_{<m}|$ of the available instantaneous side-information $s_{<m}$ increases with *m* in the broadcast case, it remains the same for the successive point-to-point protocol. Therefore, a higher cardinality $|{𝕊}_{m}|$ is required compared to the broadcast case to avoid additional compression losses.

**Algorithm 2:** Extended Blahut–Arimoto algorithm for the successive point-to-point protocol.
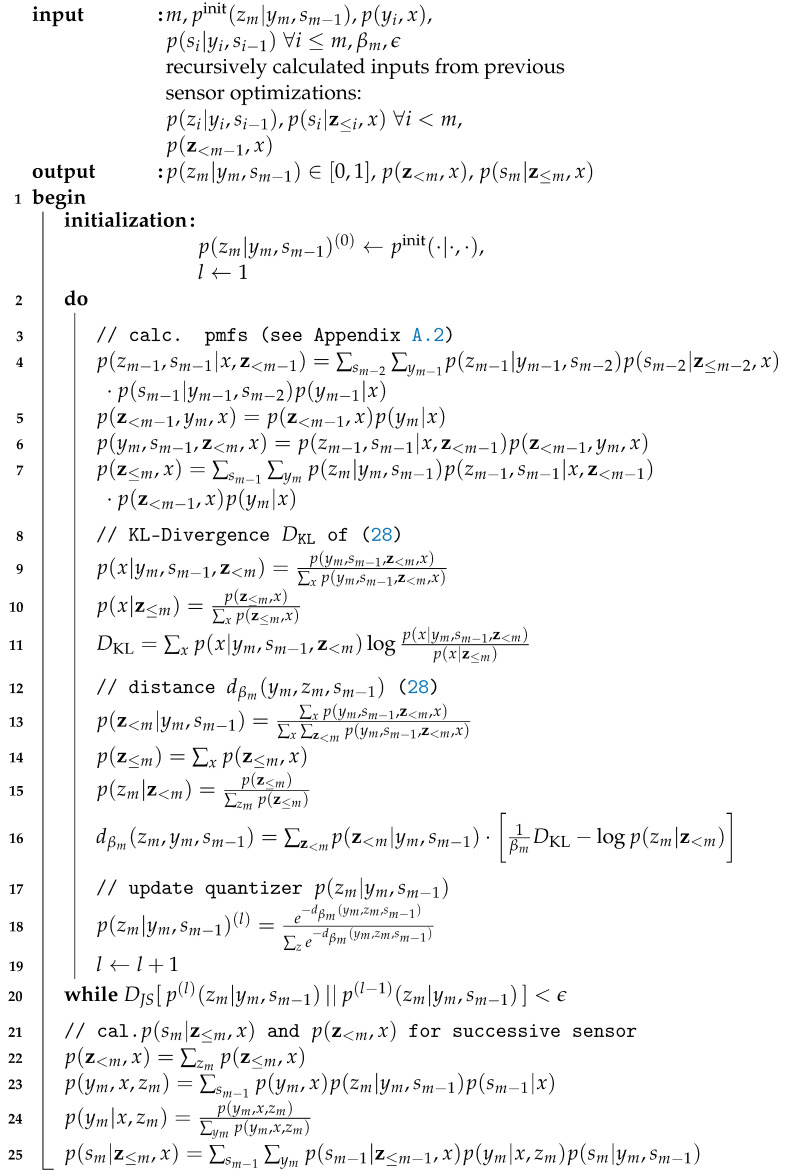


#### 5.2.4. Performance for Different Network Sizes

**Figure 13:**
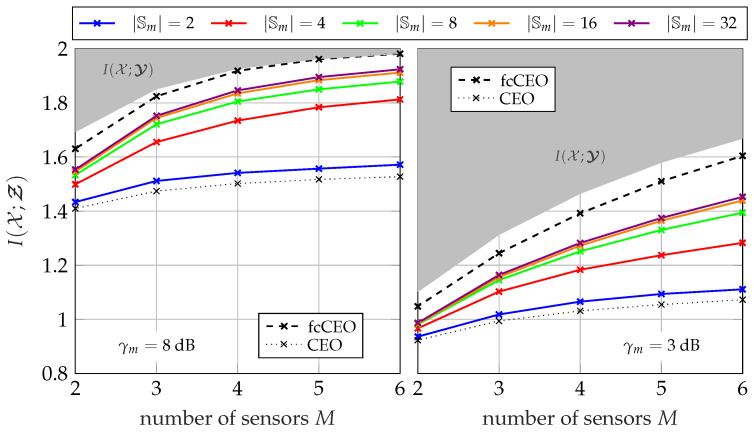
Relevant mutual information I(𝒳;Ƶ) versus the network size for a fixed sum-rate of $C_{\mathrm{sum}}=2.5$ bit/s/Hz and $C_{m}=\frac{C_{\mathrm{sum}}}{M}$ using the successive point-to-point transmission protocol with different cardinalities $|{𝕊}_{m}|$; |𝕏|=4, $|{𝕐}_{m}|\;=64$, $|{\mathbb{Z}}_{m}|\;=4$.

**Figure 14:**
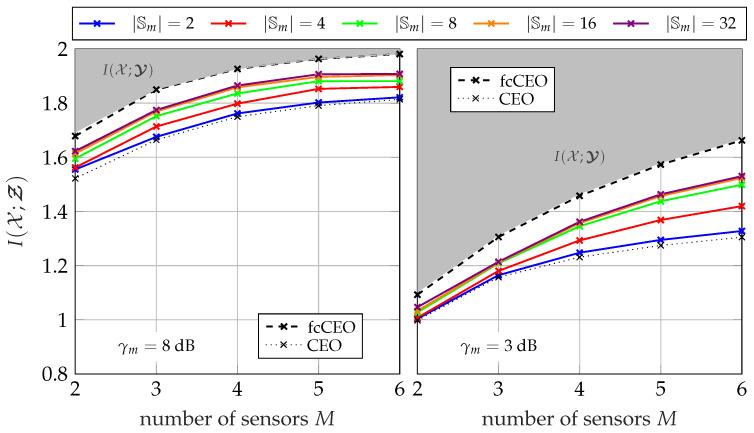
Relevant mutual information I(𝒳;Ƶ) versus the network size for a fixed sum-rate of $C_{\mathrm{sum}}=4$ bit/s/Hz and $C_{m}=\frac{C_{\mathrm{sum}}}{M}$ using the successive point-to-point transmission protocol with different cardinalities $|{𝕊}_{m}|$; |𝕏|=4, $|{𝕐}_{m}|\;=64$, $|{\mathbb{Z}}_{m}|\;=4$.

Figure 13 and Figure 14 illustrate the overall performance of the pcCEO system with point-to-point exchanged instantaneous side-information where all sensors share the same channel to the common receiver with a fixed sum-rate $C_{\mathrm{sum}}=\sum_{m=1}^{M}C_{m}$ in an orthogonal way and a round robin fashion. Again, the black curves represent the non-cooperative CEO scenario and the fcCEO scenario. Hence, they serve as lower and upper bound, respectively. In general, the curves are very similar to those for broadcasting instantaneous side-information in Figure 8 and Figure 9. Independent of the SNR or the sum-rate $C_{\mathrm{sum}}$, the relevant mutual information I(𝒳;Ƶ) increases for larger networks and even a single bit as instantaneous side-information $|{𝕊}_{m}|\;=2$ leads to slight improvements compared to the non-cooperative case. However, there still remains a gap to the fcCEO scenario even for large $|{𝕊}_{m}|$, which results from the successive communication strategy, since sensors at the beginning of the optimization chain can exploit no or little instantaneous side-information.

#### 5.2.5. Performance for Different Sum-Rates

**Figure 15:**
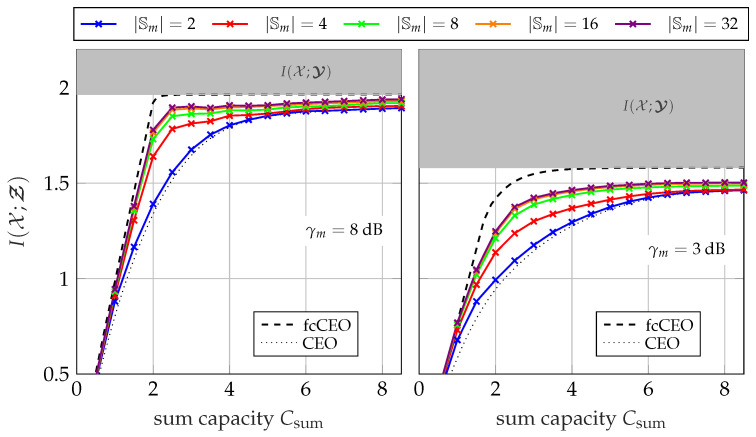
Relevant mutual information I(𝒳;Ƶ) versus sum-rate $C_{\mathrm{sum}}$ with $C_{m}=\frac{C_{\mathrm{sum}}}{M}$ using the successive point-to-point transmission protocol with different cardinalities $|{𝕊}_{m}|$; M=5, |𝕏|=4, $|{𝕐}_{m}|\;=64$, $|{\mathbb{Z}}_{m}|\;=4$.

Figure 15 illustrates the influence of the sum-rate $C_{\mathrm{sum}}=\sum_{m=1}^{M}C_{m}$ for a scenario with M=5 sensors. Naturally, larger sum-rates correlate with higher individual link capacities. Again, CEO and fcCEO scenarios provide lower and upper bounds, respectively. For a cardinality of $|{𝕊}_{m}|\;=2$, only a small gain compared to the non-cooperative CEO scenario can be observed. However, the gain gets more and more significant with increasing $|{𝕊}_{m}|$. Comparing the results to the upper fcCEO bound illuminates the loss due to limited available side-information at early transmitting sensors. The largest difference can be observed for sum-rates between $2\leq C_{\mathrm{sum}}\leq 4$ bit/s/Hz.

#### 5.2.6. Asymmetric Scenarios

A very important part is the investigation of asymmetric scenarios. As the achievable relevant information I(𝒳;Ƶ) of a non-cooperative CEO scenario is very sensitive to the optimization order, i.e., the Wyner–Ziv coding strategy, in asymmetric scenarios [14] the question arises if the exchange of instantaneous side-information can improve the robustness against bad optimization orders. Therefore, the same two asymmetric setups as in [14] are analyzed. Scenario 1 considers the case where sensors with low SNRs $\gamma_{m}$ have low link capacities $C_{m}$ while sensors with high SNRs $\gamma_{m}$ have high link capacities $C_{m}$. Scenario 2 considers the opposite case, where sensors with low SNRs have high link capacities and vice versa.

**Figure 16:**
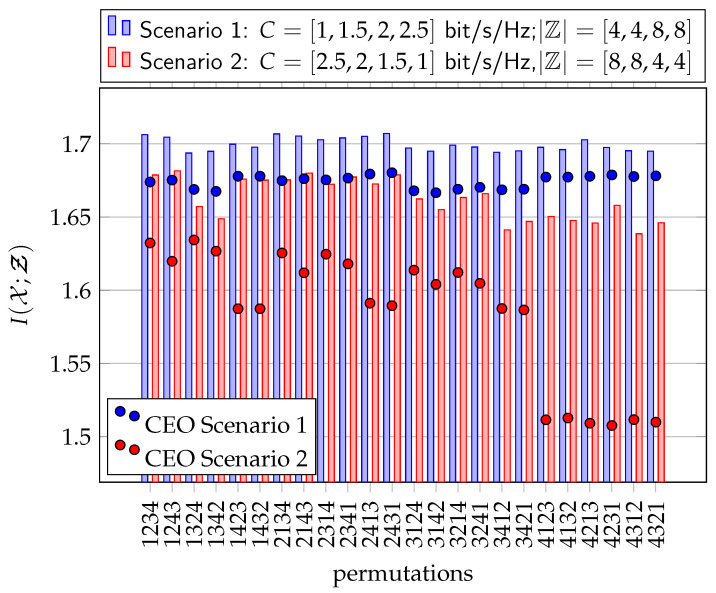
Relevant mutual information I(𝒳;Ƶ) for non-symmetric scenario with M=4 sensors, SNRs $\gamma_{m}$ = [2,4,6,8] dB and |𝕏|=4, $|{𝕐}_{m}|\;=64$, $|{\mathbb{Z}}_{m}|\;=4$ using the successive point-to-point transmission protocol with $|{𝕊}_{m}|\;=8$.

Figure 16 illustrates the relevant mutual information I(𝒳;Ƶ) for all M!=24 sensor permutations for a network of M=4 sensors. The dots represent the results from [14] for a non-cooperative CEO scenario. Blue dots show Scenario 1 while the red dots represent Scenario 2. The results for the pcCEO scenario with successive point-to-point side-information exchange is depicted as bars.

Comparing the non-cooperative case with the successive point-to-point exchange of side-information for Scenario 1, we observe a slight increase of the overall relevant mutual information I(𝒳;Ƶ) for partial cooperation and this particular scenario. Moreover, the influence of the Wyner–Ziv coding strategy (optimization order) becomes smaller due to cooperation. The performance for Scenario 2 is worse than the performance for Scenario 1, again for both the cooperative and the non-cooperative case. In this scenario, accurate measurements have to be strongly compressed in order to forward them to the common receiver while unreliable measurements cannot contribute much to the overall performance although they can be forwarded to the common receiver at high rates. However, the loss due to bad optimization orders is much lower for partial cooperation. A sensor with a bad forward channel and a high SNR can still forward its information to the next sensor, which might have a better forward channel. Therefore, exchanging instantaneous side-information can improve the robustness against bad optimization orders.

### 5.3. Two-Phase Transmission Protocol with Artificial Side-Information

Previous subsections revealed that partial cooperation by exchanging instantaneous side-information improves the overall performance. However, a gap to the fcCEO scenario still remains, and we claimed that the successive exchange of instantaneous side-information is the reason for this difference. Due to the sequential forwarding protocols considered so far, early sensors have no or little instantaneous side-information. They hardly profit from the cooperation as opposed to the full cooperation case where all sensors have access to the complete information. In order to substantiate this statement, a third transmission protocol consisting of two phases is considered. Inspired by the fcCEO scenario, the first cooperation phase is used to exchange instantaneous side-information between all sensors, while the transmission phase is used to forward the information to the common receiver in the usual way. The difference to the fcCEO scenario is that only compressed versions of the observations can be exchanged during the cooperation phase.

**Figure 17:**
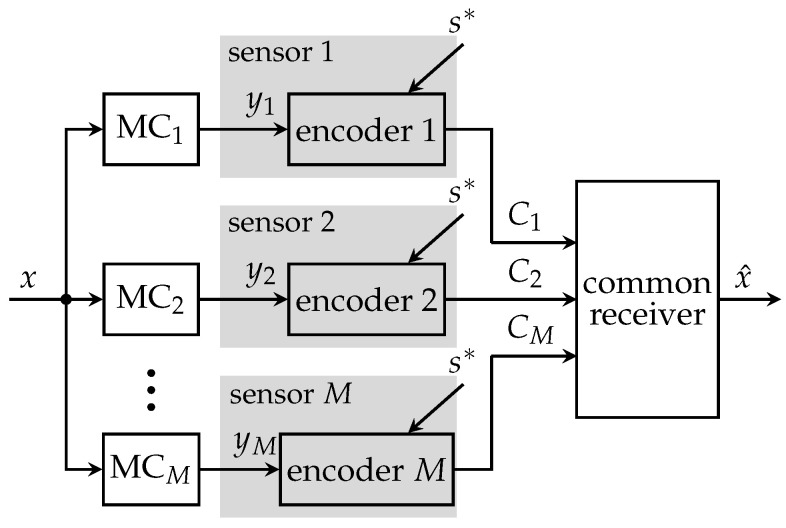
Partially cooperative CEO scenario using the two-phase transmission protocol.

For simplicity, we assume that each sensor obtains the same instantaneous side-information represented by $s^{*}$, independent of its position in the optimization chain, see Figure 17. Moreover, we pursue the EXIT chart philosophy [33], where extrinsic information is artificially created to analyze the information exchange between decoders in concatenated coding schemes. In the pcCEO context, the artificial side-information can be interpreted as extrinsic information about the relevant signal *x* being generated by adding AWGN to *x*. The noise variance is adapted to obtain a specific SNR $\gamma_{\mathrm{extr}}$ or equivalently a desired mutual side-information $I(𝒳;{𝒮}^{*})$. It has to be emphasized that $\gamma_{\mathrm{extr}}$ can be chosen independently from the measurement SNRs at the sensors in order to obtain general conclusions. Since the instantaneous side-information is created artificially, $s^{*}$ is assumed to be independent of the indexes $y_{m}$ given the relevant signal *x*, i.e., $p\left(y_{m},s^{*}|x\right)=p\left(y_{m}|x\right)p\left(s^{*}|x\right)$ holds. This simplifies the Markovian structure of the optimization problem which equals the one of the original CEO problem. With the same argumentation as in the original CEO problem, we claim that the supermodularity holds and the greedy optimization structure is optimal. This model leads to the modified optimization problem

(29) $$\begin{array}{cc} L_{\mathrm{GDIB}-\mathrm{TP}}^{\left(1\right)} & =I\left(𝒳;{Ƶ}_{1}\right)-\beta_{1}I\left({𝒴}_{1},{𝒮}^{*};{Ƶ}_{1}\right)\\ \; & \vdots \end{array}$$

(30) $$\begin{array}{cc} L_{\mathrm{GDIB}-\mathrm{TP}}^{\left(M\right)} & =I\left(𝒳;{Ƶ}_{M}|{Ƶ}_{<M}\right)-\beta_{M}I\left({𝒴}_{M},{𝒮}^{*};{Ƶ}_{M}|{Ƶ}_{<M}\right). \end{array}$$

The optimization problem can be solved using the same strategy as described in previous Section 5.1 and Section 5.2. This leads to the implicit update equation

(31) $$\begin{array}{c} p\left(z_{m}|y_{m},s^{*}\right)=\frac{e^{-d_{\beta_{m}}\left(y_{m},z_{m},s^{*}\right)}}{\sum_{z_{m}}e^{-d_{\beta_{m}}\left(y_{m},z_{m},s^{*}\right)}} \end{array}$$

with

(32) $$\begin{array}{cc} \; & \;\;d_{\beta_{m}}\left(y_{m},z_{m},s^{*}\right)≔E_{{Ƶ}_{<m}|y_{m},s^{*}}[\frac{1}{\beta_{m}}\cdot \\ \; & D_{KL}[p\left(x|y_{m},s^{*},z_{<m}\right)∥p\left(x|z_{\leq m}\right)]-\log p\left(z_{m}|z_{<m}\right)]. \end{array}$$

#### 5.3.1. Performance of Two-Phase Transmission

**Figure 18:**
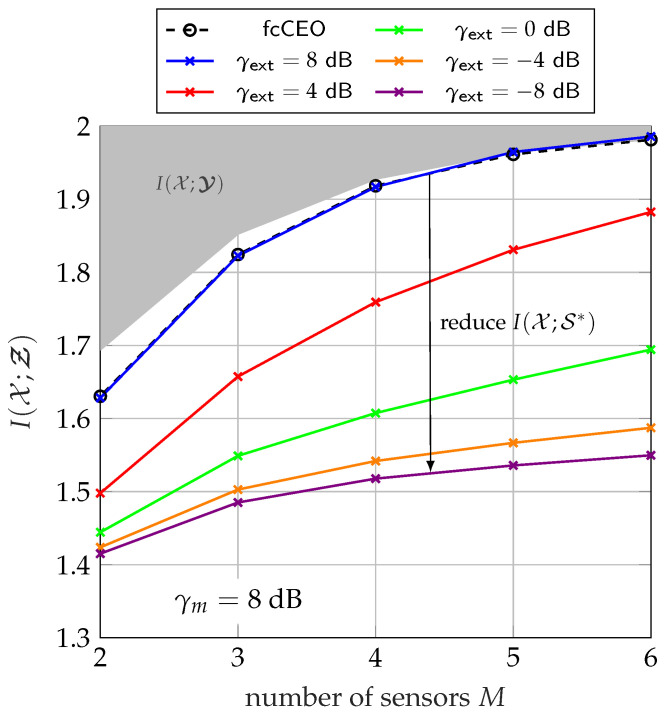
Relevant mutual information I(𝒳;Ƶ) versus the network size for a fixed sum-rate of $C_{\mathrm{sum}}=2.5$ bit/s/Hz and $C_{m}=\frac{C_{\mathrm{sum}}}{M}$ using a two-phase transmission protocol for artificially decoupled extrinsic information with different $\gamma_{\mathrm{ext}}$; $\gamma_{m}=8$ dB, |𝕏|=4, $|{𝕐}_{m}|\;=64$, $|{\mathbb{Z}}_{m}|\;=4$, $|{𝕊}^{*}|\;=512$.

Figure 18 illustrates the same experiment as in Figure 8 or Figure 13, but for the two-phase transmission protocol. The extrinsic information is chosen independent of the measurement SNR and has its own SNR $\gamma_{\mathrm{ext}}$ represented by different colors in Figure 18. The cardinality of the extrinsic information is chosen as $|{𝕊}^{*}|\;=512$ to not introduce any compression losses. As before, the black dashed line represents the fcCEO scenario. The curve for $\gamma_{\mathrm{extr}}=\gamma_{m}=8$ dB represents the case where each sensor forwards instantaneous side-information whose quality corresponds to its measurement SNR. We observe the same performance as for the fcCEO scenario. This demonstrates that the remaining performance gap to the fcCEO scenario disappears completely for appropriate cooperation among sensors. Naturally, decreasing the SNR of the extrinsic information $\gamma_{\mathrm{ext}}$ or equivalently $I(𝒳;{𝒮}^{*})$ leads to a lower overall performance I(𝒳;Ƶ).

#### 5.3.2. Influence of Extrinsic Information

**Figure 19:**
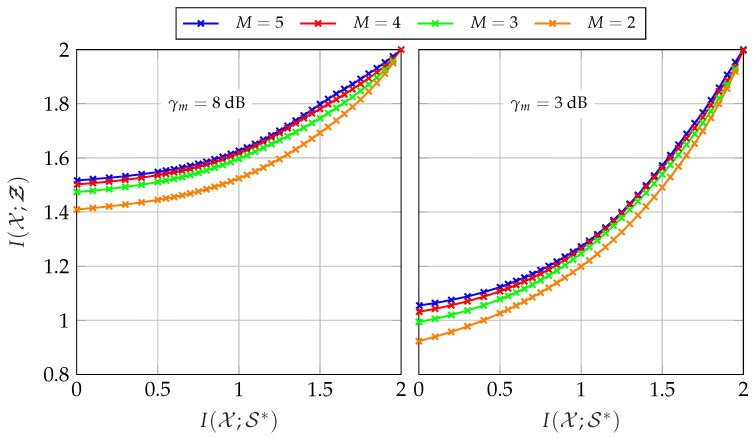
Relevant mutual information I(𝒳;Ƶ) versus extrinsic mutual information $I(𝒳;{𝒮}^{*})$ for different network sizes and a fixed sum-rate of $C_{\mathrm{sum}}=2.5$ bit/s/Hz and $C_{m}=\frac{C_{\mathrm{sum}}}{M}$ and |𝕏|=4, $|{𝕐}_{m}|\;=64$, $|{\mathbb{Z}}_{m}|\;=4$.

The influence of extrinsic information is depicted in Figure 19 for $\gamma_{m}=8$ dB and $\gamma_{m}=3$ dB. Therefore, the overall relevant mutual information I(𝒳;Ƶ) is depicted versus the mutual information of the extrinsic information $I(𝒳;{𝒮}^{*})$ for different network sizes. As before, all sensors share the same forward channel with $C_{\mathrm{sum}}=2.5$ bit/s/Hz and $C_{m}=\frac{C_{\mathrm{sum}}}{M}$. Providing no extrinsic information, i.e., $I(𝒳;{𝒮}^{*})=0$ delivers the same result as the non-cooperative CEO scenario of Section 3. Naturally, enhancing the quality of the extrinsic information increases the overall relevant mutual information I(𝒳;Ƶ) up to the maximum of 2 bit/s/Hz.

## 6. Conclusions

This paper extends the non-cooperative CEO scenario allowing partial cooperation among sensors in the network. Therefore, it extends the algorithmic solution introduced in [14] for three different inter-sensor communication protocols: successive broadcasting, successive point-to-point communication and a two-phase transmission protocol. The first two protocols perform the exchange of instantaneous side-information and forwarding information to the common receiver at the same time step. Therefore, successive broadcasting exploits the instantaneous side-information of all previous sensors within the optimization chain. Since this may cause dimensionality problems during the optimization, the successive point-to-point transmission protocol forwards the instantaneous side-information only to the next sensor. It turns out that allowing this partial communication outperforms the non-cooperative compression where no communication among sensors is possible. Moreover, cooperative compression shows a larger robustness to suboptimal Wyner–Ziv coding strategies in asymmetric scenarios. However, a small performance gap to the fcCEO scenario still remains for the proposed successive broadcasting and successive point-to-point transmission protocols. This gap can be closed by a third protocol separating the cooperation from the forwarding phase and allowing each sensor to access the maximal available side-information. Although no formal conclusion about the optimality of the pcCEO can be drawn, the closeness to the fcCEO scenario in the investigated simulations reveals that solutions found by the proposed greedy algorithms are at least close to optimal.

## Data Availability

Not applicable.

## Appendix A

### Appendix A.1. Optimization for Broadcasting Side-Information

According to Section 5.1, partial cooperation by broadcasting side-information requires the maximization of

(A1) $$\begin{array}{c} L_{\mathrm{GDIB}-\mathrm{BC}}^{\left(m\right)}=I\left(𝒳;{Ƶ}_{m}|{Ƶ}_{<m}\right)-\beta_{m}I\left({𝒴}_{m},{𝓢}_{<m};{Ƶ}_{m}|{Ƶ}_{<m}\right). \end{array}$$

The variational problem can be solved by taking the derivative with respect to the mapping $p(z_{m}|y_{m},s_{<m})$ and equating it to zero. In the following, the derivatives of both mutual information are given.

#### Appendix A.1.1. Derivative of *I*(𝒳; *Ƶ*_*m*_|***Ƶ***_<*m*_)

The relevant mutual information in (A1) can be rewritten such that the desired mapping occurs explicitly.

(A2) $$\begin{array}{cc} I(𝒳;{Ƶ}_{m}|{Ƶ}_{<m})\; & =E_{𝒳,{Ƶ}_{m},{Ƶ}_{<m}}[\log\frac{p(z_{m}|x,z_{<m})}{p(z_{m}|z_{<m})}]\\ \; & =\sum_{z_{m}}\sum_{z_{<m}}\sum_{x}\sum_{y_{m}}\sum_{s_{<m}}p\left(z_{m}|y_{m},s_{<m}\right)p\left(y_{m},s_{<m},z_{<m},x\right)\\ & \;\cdot \log\sum_{a\in {𝕐}_{m}}\sum_{b\in {𝕊}_{<m}}p\left(z_{m}|a,b\right)p\left(a,b|z_{<m},x\right)\\ \; & -\sum_{z_{m}}\sum_{z_{<m}}\sum_{x}\sum_{y_{m}}\sum_{s_{<m}}p\left(z_{m}|y_{m},s_{<m}\right)p\left(y_{m},s_{<m},z_{<m},x\right)\\ & \;\cdot \log\sum_{a\in {𝕐}_{m}}\sum_{b\in {𝕊}_{<m}}p\left(z_{m}|a,b\right)p\left(a,b|z_{<m}\right) \end{array}$$

The derivative of (A2) delivers

(A3) $$\begin{array}{cc} & \frac{\partial I(𝒳;{Ƶ}_{m}|{Ƶ}_{<m})}{\partial p(z_{m}|y_{m},s_{<m})}\\ \; & \;=\sum_{x}\sum_{z_{<m}}p\left(y_{m},s_{<m},z_{<m},x\right)\cdot \log p\left(z_{m}|x,z_{<m}\right)\\ \; & \;+\sum_{x}\sum_{z_{<m}}\underset{=p(z_{m},z_{<m},x)}{\underbrace{\left[\sum_{s_{<m}}\sum_{y_{m}}p\left(z_{m}|y_{m},s_{<m}\right)p\left(y_{m},s_{<m},z_{<m},x\right)\right]}}\cdot \frac{p(y_{m},s_{<m}|x,z_{<m})}{p(z_{m}|x,z_{<m})}\\ \; & \;-\sum_{x}\sum_{z_{<m}}p\left(y_{m},s_{<m},z_{<m},x\right)\cdot \log p\left(z_{m}|z_{<m}\right)\\ \; & \;-\sum_{x}\sum_{z_{<m}}\underset{=p(z_{m},z_{<m},x)}{\underbrace{\left[\sum_{s_{<m}}\sum_{y_{m}}p\left(z_{m}|y_{m},s_{<m}\right)p\left(y_{m},s_{<m},z_{<m},x\right)\right]}}\cdot \frac{p(y_{m},s_{<m}|z_{<m})}{p(z_{m}|z_{<m})}\\ \; & \;=\sum_{z_{<m}}p\left(y_{m},s_{<m},z_{<m}\right)\sum_{x}p\left(x|y_{m},s_{<m},z_{<m}\right)\log\frac{p(z_{m}|x,z_{<m})}{p(z_{m}|z_{<m})}. \end{array}$$

Exploiting Bayes’ theorem, the argument of the logarithmic function can be rewritten

(A4) $$\begin{array}{cc} \frac{p(z_{m}|x,z_{<m})}{p(z_{m}|z_{<m})}\; & =\frac{p(x|z_{\leq m})}{p(x|z_{<m})}\\ \; & =\frac{p(x|z_{\leq m})}{p(x|y_{m},s_{<m},z_{<m})}\cdot \frac{p(x|y_{m},s_{<m},z_{<m})}{p(x|z_{<m})}. \end{array}$$

The last ratio in (A4) can be dropped because it does not depend on $p(z_{m}|y_{m},s_{<m})$ and its contribution can be incorporated into the Lagrange multiplier $\beta_{m}$. The insertion of the first ratio into (A3) yields the contribution of the derivative of the relevant mutual information

(A5) $$\begin{array}{cc} \frac{\partial I(𝒳;{Ƶ}_{m}|{Ƶ}_{<m})}{\partial p(z_{m}|y_{m},s_{<m})}\; & \to -\sum_{z_{<m}}p\left(y_{m},s_{<m},z_{<m}\right)\sum_{x}p\left(x|y_{m},s_{<m},z_{<m}\right)\log\frac{p(x|y_{m},s_{<m},z_{<m})}{p(x|z_{\leq m})}\\ \; & =-\sum_{z_{<m}}p\left(y_{m},s_{<m},z_{<m}\right)D_{KL}[p\left(x|y_{m},s_{<m},z_{<m}\right)∥p\left(x|z_{\leq m}\right)]. \end{array}$$

#### Appendix A.1.2. Derivative of *I*(𝒴_*m*_, ***𝓢***_<*m*_; *Ƶ_m_*|***Ƶ***_<*m*_)

With the definition of the conditional compression rate

(A6) $$\begin{array}{cc} & I\left({𝒴}_{m},{𝓢}_{<m};{Ƶ}_{m}|{Ƶ}_{<m}\right)=E_{{𝒴}_{m},{Ƶ}_{m},{Ƶ}_{<m},{𝓢}_{<m}}[\log\frac{p(z_{m}|y_{m},s_{<m},z_{<m})}{p(z_{m}|z_{<m})}]\\ & =\sum_{z_{m}}\sum_{z_{<m}}\sum_{s_{<m}}\sum_{y_{m}}p\left(z_{m}|y_{m},s_{<m}\right)p\left(y_{m},s_{<m},z_{<m}\right)\log p\left(z_{m}|y_{m},s_{<m}\right)\\ \; & -\sum_{z_{m}}\sum_{z_{<m}}\sum_{s_{<m}}\sum_{y_{m}}p\left(z_{m}|y_{m},s_{<m}\right)p\left(y_{m},s_{<m},z_{<m}\right)\log\sum_{a\in {𝕐}_{m}}\sum_{b\in {𝕊}_{<m}}p\left(z_{m}|a,b\right)p\left(a,b|z_{<m}\right) \end{array}$$

its derivative becomes

(A7) $$\begin{array}{cc} & \frac{\partial I({𝒴}_{m},{𝓢}_{<m};{Ƶ}_{m}|{Ƶ}_{<m})}{\partial p(z_{m}|y_{m},s_{<m})}\\ \; & \;=\sum_{z_{<m}}p\left(y_{m},s_{<m},z_{<m}\right)\log p\left(z_{m}|y_{m},s_{<m}\right)\\ & \;+\sum_{z_{<m}}p\left(y_{m},s_{<m},z_{<m}\right)\frac{p(z_{m}|y_{m},s_{<m})}{p(z_{m}|y_{m},s_{<m})}\\ \; & \;-\sum_{z_{<m}}p\left(y_{m},s_{<m},z_{<m}\right)\log p\left(z_{m}|z_{<m}\right)\\ \; & \;-\sum_{z_{<m}}\underset{=p(z_{m},z_{<m})}{\underbrace{\left[\sum_{y_{m}}\sum_{s_{<m}}p\left(z_{m}|y_{m},s_{<m}\right)p\left(y_{m},s_{<m},z_{<m}\right)\right]}}\cdot \frac{p(y_{m},s_{<m}|z_{<m})}{p(z_{m}|z_{<m})}\\ \; & \;=\sum_{z_{<m}}p\left(y_{m},s_{<m},z_{<m}\right)\log\frac{p(z_{m}|y_{m},s_{<m})}{p(z_{m}|z_{<m})}. \end{array}$$

#### Appendix A.1.3. Fusion of Derived Parts

Combining the result in (A5) and (A7) delivers the complete derivative

(A8) $$\begin{array}{cc} & -\sum_{z_{<m}}p\left(y_{m},s_{<m},z_{<m}\right)D_{KL}[p\left(x|y_{m},s_{<m},z_{<m}\right)∥p\left(x|z_{\leq m}\right)]\\ \; & -\;\beta_{m}\sum_{z_{<m}}p\left(y_{m},s_{<m},z_{<m}\right)\log p\left(z_{m}|y_{m},s_{<m}\right)\\ & +\;\beta_{m}\sum_{z_{<m}}p\left(y_{m},s_{<m},z_{<m}\right)\cdot \log p\left(z_{m}|z_{<m}\right)=0. \end{array}$$

Following the idea of Blahut and Arimoto [34], $p(x|z_{\leq m})$ and $p(z_{m}|z_{<m})$ are assumed to be independent of $p(z_{m}|y_{m},s_{<m})$. With this trick, (A8) can be resolved with respect to the desired mapping of sensor *m* leading to the implicit solution

(A9) $$p\left(z_{m}|y_{m},s_{<m}\right)=\frac{e^{-d_{\beta_{m}}\left(y_{m},z_{m},s_{<m}\right)}}{\sum_{z_{m}}e^{-d_{\beta_{m}}\left(y_{m},z_{m},s_{<m}\right)}}$$

with

(A10) $$\begin{array}{cc} \; & d_{\beta_{m}}\left(y_{m},z_{m},s_{<m}\right)\\ \; & \;≔\sum_{z_{<m}}p\left(z_{<m}|y_{m},s_{<m}\right)\left[\frac{1}{\beta_{m}}D_{KL}[p\left(x|y_{m},s_{<m},z_{<m}\right)∥p\left(x|z_{\leq m}\right)]-\log p\left(z_{m}|z_{<m}\right)\right]\\ \; & \;=E_{{Ƶ}_{<m}|y_{m},s_{<m}}\left[\frac{1}{\beta_{m}}D_{KL}[p\left(x|y_{m},s_{<m},z_{<m}\right)∥p\left(x|z_{\leq m}\right)]-\log p\left(z_{m}|z_{<m}\right)\right]. \end{array}$$

#### Appendix A.1.4. Calculating Required pmfs

This section covers the calculation of the required pmfs for the previously described algorithm. The first term in the KL divergence in (A10) is defined by

(A11) $$\begin{array}{c} p\left(x|y_{m},s_{<m},z_{<m}\right)=\frac{p(x,y_{m},s_{<m},z_{<m})}{\sum_{x}p\left(x,y_{m},s_{<m},z_{<m}\right)} \end{array}$$

where $p(x,y_{m},s_{<m},z_{<m})$ is determined recursively as

(A12) $$\begin{array}{cc} & p\left(x,y_{m},s_{<m},z_{<m}\right)=\sum_{y_{m-1}}p\left(z_{m-1}|y_{m-1},s_{<m-1}\right)\cdot \\ & \;p\left(s_{m-1}|y_{m-1},s_{<m-1}\right)p\left(y_{m}|x\right)p\left(x,y_{m-1},s_{<m-1},z_{<m-1}\right). \end{array}$$

In (A12), the pmf $p(z_{m-1}|y_{m-1},s_{<m-1})$ represents the quantizer mapping of the previous sensor m−1 while $p(s_{m-1}|y_{m-1},s_{<m-1})$ denotes its the mapping for the instantaneous side-information. Hence, both have already been determined when optimizing sensor m−1 leading to a recursive computation. The second term in the KL divergence in (A10) is calculated by

(A13) $$\begin{array}{c} p\left(x|z_{\leq m}\right)=\frac{p(z_{\leq m},x)}{\sum_{x}p\left(z_{\leq m},x\right)} \end{array}$$

where $p(z_{\leq m},x)$ can be calculated by

(A14) $$\begin{array}{c} p\left(z_{\leq m},x\right)=\sum_{s_{<m}}\sum_{y_{m}}p\left(z_{m}|y_{m},s_{<m}\right)p\left(x,y_{m},s_{<m},z_{<m}\right) \end{array}$$

with $p(z_{m}|y_{m},s_{<m})$ being the quantizer mapping of the current sensor. The joint pmf $p(x,y_{m},s_{<m},z_{<m})$ has already been calculated in (A12). The argument of the logarithm in (A10) can be derived as

(A15) $$\begin{array}{c} p\left(z_{m}|z_{<m}\right)=\frac{\sum_{x}p\left(z_{\leq m},x\right)}{\sum_{z_{m}}\sum_{x}p\left(z_{\leq m},x\right)}. \end{array}$$

Finally, the pmf to calculate the conditional expectation in (A10) is determined as

(A16) $$\begin{array}{c} p\left(z_{<m}|y_{m},s_{<m}\right)=\frac{\sum_{x}p\left(x,y_{m},s_{<m},z_{<m}\right)}{\sum_{z_{<m}}\sum_{x}p\left(x,y_{m},s_{<m},z_{<m}\right)}\;. \end{array}$$

Note that all above equations simplify to the scalar IB equations given in Section 2 when optimizing the first sensor.

### Appendix A.2. Optimization for Point-to-Point Exchange of Side-Information

According to Section 5.2, partial cooperation using a point-to-point communication protocol requires the maximization of

(A17) $$\begin{array}{c} L_{\mathrm{GDIB}-\mathrm{PTP}}^{\left(m\right)}=I\left(𝒳;{Ƶ}_{m}|{Ƶ}_{<m}\right)-\beta_{m}I\left({𝒴}_{m},{𝒮}_{m-1};{Ƶ}_{m}|{Ƶ}_{<m}\right). \end{array}$$

The variational problem can be solved by taking the derivative with respect to the mapping $p(z_{m}|y_{m},s_{m-1})$ and equating it to zero. In the following, the derivatives of both mutual information are given.

#### Appendix A.2.1. Derivative of *I*(𝒳; *Ƶ_m_*|***Ƶ***_<*m*_)

The relevant mutual information in (A17) can be rewritten such that the desired mapping occurs explicitly.

(A18) $$\begin{array}{cc} I(𝒳;{Ƶ}_{m}|{Ƶ}_{<m})\; & =E_{𝒳,{Ƶ}_{m},{Ƶ}_{<m}}\left[\log\frac{p(z_{m}|x,z_{<m})}{p(z_{m}|z_{<m})}\right]\\ \; & =\sum_{z_{\leq m}}\sum_{x}\sum_{y_{m}}\sum_{s_{m-1}}p\left(z_{m}|y_{m},s_{m-1}\right)p\left(y_{m},s_{m-1},z_{<m},x\right)\\ & \;\cdot \log\sum_{a\in {𝕐}_{m}}\sum_{b\in {𝕊}_{m-1}}p\left(z_{m}|a,b\right)p\left(a,b|z_{<m},x\right)\\ \; & -\sum_{z_{\leq m}}\sum_{x}\sum_{y_{m}}\sum_{s_{m-1}}p\left(z_{m}|y_{m},s_{m-1}\right)p\left(y_{m},s_{m-1},z_{<m},x\right)\\ & \;\cdot \log\sum_{a\in {𝕐}_{m}}\sum_{b\in {𝕊}_{m-1}}p\left(z_{m}|a,b\right)p\left(a,b|z_{<m}\right) \end{array}$$

The derivative of (A18) delivers

(A19) $$\begin{array}{cc} & \frac{\partial I(𝒳;{Ƶ}_{m}|{Ƶ}_{<m})}{\partial p(z_{m}|y_{m},s_{m-1})}\\ \; & =\sum_{x}\sum_{z_{<m}}p\left(y_{m},s_{m-1},z_{<m},x\right)\cdot \log p\left(z_{m}|x,z_{<m}\right)\\ \; & +\sum_{x}\sum_{z_{<m}}\underset{=p(z_{m},z_{<m},x)}{\underbrace{\left[\sum_{s_{m-1}}\sum_{y_{m}}p\left(z_{m}|y_{m},s_{m-1}\right)p\left(y_{m},s_{m-1},z_{<m},x\right)\right]}}\cdot \frac{p(y_{m},s_{m-1}|x,z_{<m})}{p(z_{m}|x,z_{<m})}\\ \; & -\sum_{x}\sum_{z_{<m}}p\left(y_{m},s_{m-1},z_{<m},x\right)\cdot \log p\left(z_{m}|z_{<m}\right)\\ \; & -\sum_{x}\sum_{z_{<m}}\underset{=p(z_{m},z_{<m},x)}{\underbrace{\left[\sum_{s_{m-1}}\sum_{y_{m}}p\left(z_{m}|y_{m},s_{m-1}\right)p\left(y_{m},s_{m-1},z_{<m},x\right)\right]}}\cdot \frac{p(y_{m},s_{m-1}|z_{<m})}{p(z_{m}|z_{<m})}\\ \; & =\sum_{x}\sum_{z_{<m}}p\left(y_{m},s_{m-1},z_{<m},x\right)\cdot \log\frac{p(z_{m}|x,z_{<m})}{p(z_{m}|z_{<m})}\\ \; & =\sum_{z_{<m}}p\left(y_{m},s_{m-1},z_{<m}\right)\sum_{x}p\left(x|y_{m},s_{m-1},z_{<m}\right)\log\frac{p(z_{m}|x,z_{<m})}{p(z_{m}|z_{<m})}. \end{array}$$

Exploiting Bayes’ theorem the argument of the logarithmic function can be rewritten

(A20) $$\begin{array}{cc} \frac{p(z_{m}|x,z_{<m})}{p(z_{m}|z_{<m})}\; & =\frac{p(x|z_{\leq m})}{p(x|z_{<m})}\\ \; & =\frac{p(x|z_{\leq m})}{p(x|y_{m},s_{m-1},z_{<m})}\frac{p(x|y_{m},s_{m-1},z_{<m})}{p(x|z_{<m})}. \end{array}$$

The last ratio in (A20) can be dropped because it does not depend on $p(z_{m}|y_{m},s_{m-1})$ and its contribution can be incorporated into the Lagrange multiplier $\beta_{m}$. The insertion of the first ratio into (A19) yields the contribution of the derivative of the relevant mutual information

(A21) $$\begin{array}{cc} \frac{\partial I(𝒳;{Ƶ}_{m}|{Ƶ}_{<m})}{\partial p(z_{m}|y_{m},s_{m-1})} & \to -\sum_{z_{<m}}p\left(y_{m},s_{m-1},z_{<m}\right)\sum_{x}p\left(x|y_{m},s_{m-1},z_{<m}\right)\log\frac{p(x|y_{m},s_{m-1},z_{<m})}{p(x|z_{\leq m})}\\ \; & =-\sum_{z_{<m}}p\left(y_{m},s_{m-1},z_{<m}\right)D_{KL}[p\left(x|y_{m},s_{m-1},z_{<m}\right)∥p\left(x|z_{\leq m}\right)]. \end{array}$$

#### Appendix A.2.2. Derivative of *I*(*𝒴_m_*, *𝒮*_*m*−1_; *Ƶ_m_*|***Ƶ***_<*m*_)

With the definition of the conditional compression rate

(A22) $$\begin{array}{cc} & I\left({𝒴}_{m},{𝒮}_{m-1};{Ƶ}_{m}|{Ƶ}_{<m}\right)=E_{{𝒴}_{m},{Ƶ}_{m},{Ƶ}_{<m},{𝒮}_{m-1}}[\log\frac{p(z_{m}|y_{m},s_{m-1},z_{<m})}{p(z_{m}|z_{<m})}]\\ & =\sum_{z_{m}}\sum_{z_{<m}}\sum_{s_{m-1}}\sum_{y_{m}}p\left(z_{m}|y_{m},s_{m-1}\right)p\left(y_{m},s_{m-1},z_{<m}\right)\log p\left(z_{m}|y_{m},s_{m-1}\right)\\ \; & -\sum_{z_{m}}\sum_{z_{<m}}\sum_{s_{m-1}}\sum_{y_{m}}p\left(z_{m}|y_{m},s_{m-1}\right)p\left(y_{m},s_{m-1},z_{<m}\right)\log\sum_{a\in {𝕐}_{m}}\sum_{b\in {𝕊}_{m-1}}p\left(z_{m}|a,b\right)p\left(a,b|z_{<m}\right) \end{array}$$

its derivative becomes

(A23) $$\begin{array}{cc} & \frac{\partial I({𝒴}_{m},{𝒮}_{m-1};{Ƶ}_{m}|{Ƶ}_{<m})}{\partial p(z_{m}|y_{m},s_{m-1})}\\ \; & \;=\sum_{z_{<m}}p\left(y_{m},s_{m-1},z_{<m}\right)\log p\left(z_{m}|y_{m},s_{m-1}\right)\\ & \;+\sum_{z_{<m}}p\left(y_{m},s_{m-1},z_{<m}\right)\frac{p(z_{m}|y_{m},s_{m-1})}{p(z_{m}|y_{m},s_{m-1})}\\ \; & \;-\sum_{z_{<m}}p\left(y_{m},s_{m-1},z_{<m}\right)\log p\left(z_{m}|z_{<m}\right)\\ \; & \;-\sum_{z_{<m}}\underset{=p(z_{m},z_{<m})}{\underbrace{\left[\sum_{y_{m}}\sum_{s_{m-1}}p\left(z_{m}|y_{m},s_{m-1}\right)p\left(y_{m},s_{m-1},z_{<m}\right)\right]}}\cdot \frac{p(y_{m},s_{m-1}|z_{<m})}{p(z_{m}|z_{<m})}\\ \; & \;=\sum_{z_{<m}}p\left(y_{m},s_{m-1},z_{<m}\right)\log\frac{p(z_{m}|y_{m},s_{m-1})}{p(z_{m}|z_{<m})}. \end{array}$$

#### Appendix A.2.3. Fusion of Derived Parts

Combining the result in (A21) and (A23) delivers the complete derivative

(A24) $$\begin{array}{cc} \; & -\sum_{z_{<m}}p\left(y_{m},s_{m-1},z_{<m}\right)D_{KL}[p\left(x|y_{m},s_{m-1},z_{<m}\right)∥p\left(x|z_{\leq m}\right)]\\ \; & -\;\beta_{m}\sum_{z_{<m}}p\left(y_{m},s_{m-1},z_{<m}\right)\log p\left(z_{m}|y_{m},s_{m-1}\right)\\ & +\;\beta_{m}\sum_{z_{<m}}p\left(y_{m},s_{m-1},z_{<m}\right)\cdot \log p\left(z_{m}|z_{<m}\right)=0. \end{array}$$

Following the idea of Blahut and Arimoto [34], $p(x|z_{\leq m})$ and $p(z_{m}|z_{<m})$ are assumed to be independent of $p(z_{m}|y_{m},s_{m-1})$. With this trick, (A24) can be resolved with respect to the desired mapping of sensor *m* leading to the implicit solution

(A25) $$p\left(z_{m}|y_{m},s_{m-1}\right)=\frac{e^{-d_{\beta_{m}}\left(y_{m},z_{m},s_{m-1}\right)}}{\sum_{z_{m}}e^{-d_{\beta_{m}}\left(y_{m},z_{m},s_{m-1}\right)}}$$

with

(A26) $$\begin{array}{cc} \; & d_{\beta_{m}}\left(y_{m},z_{m},s_{m-1}\right)\\ & \;≔\sum_{z_{<m}}p\left(z_{<m}|y_{m},s_{m-1}\right)\left[\frac{1}{\beta_{m}}D_{KL}[p\left(x|y_{m},s_{m-1},z_{<m}\right)∥p\left(x|z_{\leq m}\right)]-\log p\left(z_{m}|z_{<m}\right)\right]\\ \; & \;=E_{{Ƶ}_{<m}|y_{m},s_{m-1}}\left[\frac{1}{\beta_{m}}D_{KL}[p\left(x|y_{m},s_{m-1},z_{<m}\right)∥p\left(x|z_{\leq m}\right)]-\log p\left(z_{m}|z_{<m}\right)\right]. \end{array}$$

#### Appendix A.2.4. Calculating Required pmfs

This section covers the calculation of the required pmfs for the previously described algorithm. The first term in the KL divergence in (A26) is defined by

(A27) $$\begin{array}{c} p\left(x|y_{m},s_{m-1},z_{<m}\right)=\frac{p(x,y_{m},s_{m-1},z_{<m})}{\sum_{x}p\left(x,y_{m},s_{m-1},z_{<m}\right)} \end{array}$$

with

(A28) $$\begin{array}{cc} p(x,y_{m},s_{m-1},z_{<m}) & =p\left(z_{m-1},s_{m-1}|x,z_{<m-1}\right)p\left(x,y_{m},z_{<m-1}\right). \end{array}$$

The first term on the right hand side in (A28) can be calculated by

(A29) $$\begin{array}{c} p\left(z_{m-1},s_{m-1}|x,z_{<m-1}\right)=\sum_{y_{m-1}}\sum_{s_{m-2}}p\left(z_{m-1}|y_{m-1},s_{m-2}\right)\\ \;p\left(s_{m-1}|y_{m-1},s_{m-2}\right)p\left(y_{m-1}|x\right)p\left(s_{m-2}|x,z_{<m-1}\right) \end{array}$$

where $p(z_{m-1}|y_{m-1},s_{m-2})$ represents the mapping of previously designed quantizer m−1, $p(s_{m-1}|y_{m-1},s_{m-2})$ denotes its mapping of instantaneous side-information and $p(y_{m-1}|x)$ statistically describes the corresponding known measurement channel. The last term $p(s_{m-2}|x,z_{<m-1})$ has already been calculated when optimizing previous sensors (in this case the pre-predecessor) in a recursive way

(A30) $$\begin{array}{cc} p(s_{m}|x,z_{<m}) & =\sum_{y_{m}}\sum_{s_{m-1}}p\left(s_{m}|y_{m},s_{m-1}\right)p\left(y_{m}|x,z_{m}\right)p\left(s_{m-1}|x,z_{<m-1}\right) \end{array}$$

with

(A31) $$\begin{array}{c} p\left(y_{m}|x,z_{m}\right)=\frac{p(y_{m},x,z_{m})}{\sum_{y_{m}}p\left(y_{m},x,z_{m}\right)} \end{array}$$

and

(A32) $$\begin{array}{cc} p(y_{m},x,z_{m}) & =\sum_{s_{m-1}}p\left(z_{m}|y_{m},s_{m-1}\right)p\left(y_{m}|x\right)p\left(s_{m-1}|x\right)p\left(x\right). \end{array}$$

The second term on the right hand side in (A28) can be determined by

(A33) $$\begin{array}{c} p\left(x,y_{m},z_{<m-1}\right)=p\left(y_{m}|x\right)p\left(z_{<m-1},x\right). \end{array}$$

Again, the last term in (A33) is obtained as side product when optimizing the previous sensor by

(A34) $$\begin{array}{cc} p(z_{\leq m},x) & =\sum_{y_{m}}\sum_{s_{m-1}}p\left(z_{m}|y_{m},s_{m-1}\right)p\left(z_{m-1},s_{m-1}|x,z_{<m-1}\right)p\left(x,y_{m},z_{<m-1}\right) \end{array}$$

where $p(z_{m-1},s_{m-1}|x,z_{<m-1})$ and $p(x,y_{m},z_{<m-1})$ are already defined in (A29) and (A33), respectively. The second term in the KL divergence in (A26) is calculated by

(A35) $$\begin{array}{c} p\left(x|z_{\leq m}\right)=\frac{p(z_{\leq m},x)}{\sum_{x}p\left(z_{\leq m},x\right)} \end{array}$$

where $p(z_{\leq m},x)$ is already defined in (A34). The term in the logarithm in (A26) can be expressed as

(A36) $$\begin{array}{c} p\left(z_{m}|z_{<m}\right)=\frac{\sum_{x}p\left(z_{\leq m},x\right)}{\sum_{x}\sum_{z_{m}}p\left(z_{\leq m},x\right)}. \end{array}$$

Finally, the required pmf to calculate the conditional expectation in (A26) can be determined by

(A37) $$\begin{array}{c} p\left(z_{<m}|y_{m},s_{m-1}\right)=\frac{\sum_{x}p\left(x,y_{m},s_{m-1},z_{<m}\right)}{\sum_{x}\sum_{z_{<m}}p\left(x,y_{m},s_{m-1},z_{<m}\right)} \end{array}$$

with $p(x,y_{m},s_{m-1},z_{<m})$ being already defined in (A28). Note that all above equations simplify to the scalar IB equations given in Section 2 when optimizing the first sensor. Moreover, when optimizing the second sensor, there is no pre-predecessor m−2 and its impact on the above equations can be omitted.
